# Integrated network pharmacology and experimental analysis unveil multi-targeted effect of 18α- glycyrrhetinic acid against non-small cell lung cancer

**DOI:** 10.3389/fphar.2022.1018974

**Published:** 2022-10-12

**Authors:** Rasha Irshad, Nafis Raj, Gamal A. Gabr, Nikhat Manzoor, Mohammad Husain

**Affiliations:** ^1^ Virology and Oncology Lab, Department of Biotechnology, Jamia Millia Islamia, New Delhi, India; ^2^ Medical Mycology Lab, Department of Biosciences, Jamia Millia Islamia, New Delhi, India; ^3^ Department of Pharmacology and Toxicology, College of Pharmacy, Prince Sattam Bin Abdulaziz University, Al- Kharj, Saudi Arabia

**Keywords:** 18α-Glycyrrhetinic acid, triterpenoid, NSCLC, network pharmacology, apoptosis, EGFR-PI3K/AKT pathway, survival analysis

## Abstract

Non-small cell lung cancer (NSCLC) is one of the most malignant types of cancer with soaring incidence rates worldwide, attributed to its heterogeneity and complex etiology. Evidently, alternative anti-cancer therapies comprising traditional medicines and natural products have gained attention for their ability to act as chemopreventive agents with minimal toxicities, either alone or in combination. Accumulating studies have substantiated the inevitability of network pharmacology studies for effectively mapping molecular targets of natural products against multifaceted diseases, including cancer. The 18α-Glycyrrhetinic acid (18α-GA), a triterpenoid found in licorice plants, has shown promising medicinal properties, although, its mechanism of action against NSCLC yet remains elusive. The present study was conducted to explore the anti- NSCLC potential of 18α-GA, employing integrative network pharmacology, molecular docking, and experimental research. Initially, network analysis revealed 181 common targets of 18α-GA in NSCLC as shown in the “compound-target- disease” network employing *Cytoscape 3.8.2*. Further analyses identified EGFR, AKT1, PI3KR1, MAPK1, IGF1, and SRC as the most crucial hub targets of 18α-GA against NSCLC. Moreover, molecular docking simulations and functional enrichment analyses indicated the involvement of multiple signaling pathways in suppressing NSCLC. Subsequent *in-vitro* studies verified the antiproliferative effect of 18α-GA on two NSCLC cancer cell lines, H1299 and A549. Mechanistically, 18α-GA arrested cell cycle at the G1 phase, induced apoptosis, decreased migratory potential, and protein expression levels of EGFR-PI3K/AKT, as examined by flow cytometry, morphological assessment, RT-PCR, and western blot. In conclusion, this study delineates the therapeutic potential and underlying mechanism(s) of 18α-GA as a putative novel drug against NSCLC. However, further studies are warranted to elucidate the complete molecular mechanism(s) using animal models of NSCLC.

## 1 Introduction

NSCLC is the most frequently diagnosed type of lung cancer worldwide comprising approximately 85% of all new cases with a mortality rate of 18% (Sung et al., 2021). The absence of clinical symptoms and inadequate prognosis eventually cause a delay in diagnosis, which impedes the therapeutic success of the patients. Owing to its heterogeneous nature, the treatment regime of NSCLC is personalized based on cell type, molecular profile, stage of disease and ranges from lobectomy, adjuvant immunotherapy, radiotherapy to standard platinum-based chemotherapy (Gridelli et al., 2015) However, severe toxicity, resistance, frequent recurrence, and metastasis are associated with less than five years of overall survival rate rendering these curative treatment options insufficient. Therefore, cancer therapy has been continually evolving toward its prevention in recent years, and alternative therapeutic strategies are gaining attention in lowering cancer initiation and progression rates. In this pursuit, the development of complementary strategies including the use of phytochemicals has been envisioned due to their low toxicity, effectiveness, diverse structure, affordability, and multi-targeted impact (Mustafa et al., 2021). The preventive prospects of natural products are multidimensional and can be utilized for targeting cancer in multiple ways. This aids in coping with the heterogeneity that exists among cancer cells of the same type and contributes to the restriction of tumor growth. In this direction, several phytochemicals are being routinely examined for their anti-NSCLC potential, either as a stand-alone drug or as adjuvants to standard chemotherapy.

The 18α-Glycyrrhetinic acid (18α-GA) is one such bioactive triterpenoid compound isolated from licorice (*Glycyrrhiza sp.*) roots, a perennial herb found in warm countries like India, other parts of Asia, and the Mediterranean region (Tiboni et al., 2020). Licorice is approved by the FDA to be used as a flavoring agent. The 18α-stereoisomeric form of aglycone glycyrrhetinic acid (18α-GA) is metabolically formed by partial hydrolysis of glucuronidase present in glycyrrhizin which is the primary metabolite of licorice plants (Ha et al., 1991). It demonstrates remarkable antioxidant, anti-tumor (Shetty et al., 2011), anti-viral, anti-inflammatory, hepatoprotective, and ulcer healing properties (Zong et al., 2013; Li et al., 2020). The 18α-GA is reported to have inhibited cancer cell progression in various cancer types by eliciting modifications like cell cycle arrest (Sathya et al., 2014), apoptotic induction (Pirzadeh et al., 2014), and restriction of epithelial-mesenchymal transition among others (Takeuchi et al., 2016). It has been illustrated to deregulate the Beclin-1/Bcl-2 complex and induce apoptosis *via* inhibition of MAP Kinases in neuroblastoma cells (Rahman et al., 2016). In addition, 18α-GA was shown to suppress hepatic fibrosis and subsequent suppression of hepatocellular carcinoma by modulating the TGF-β1/Smad Signaling Pathway (Zong et al., 2012). Even though a few studies have been carried out on elucidating the mechanism of action of 18α-GA against cancer, it is still in infancy, particularly in NSCLC, and needs to be evaluated for reaping its wide-ranging pharmacological advantages.

Recently, network pharmacology has emerged as a holistic tool to unveil the putative molecular bio-targets, signaling pathway intricacies, and protein-protein interactions of natural compounds/medicinal plants against various diseases by utilizing extensive databases, pharmaco-mapping, and computational strategies (Chandran et al., 2017). It has been successfully employed to describe the molecular mechanisms of various phytochemicals and drugs in targeting various diseases. Therefore, in this study, we aimed to unveil the mechanism of action and the molecular pathways affected by 18α-GA against NSCLC by network pharmacology, which was further corroborated by molecular docking studies, and the core target genes were identified. We also evaluated the potential of 18α-GA in regulating various hallmarks/phenotypes of NSCLC through *in-vitro* validation on two NSCLC cell lines.

Our findings highlight the multi-targeted effect of 18α-GA in reducing NSCLC tumorigenesis as seen by network pharmacological studies. Further, it provides evidence that 18α-GA treatment suppresses cell proliferation, induces G1 phase cell cycle arrest, represses metastasis, exhibits apoptotic cell death, and regulates EGFR- PI3K/AKT signaling in NSCLC cells. The framework of this study is described in Supplementary Figure S1.

## 2 Materials and methods

### 2.1 Bioinformatics study

#### 2.1.1 Evaluation of pharmacokinetics and physicochemical parameters of 18-αGA

The canonical SMILES of 18α-GA were input into SwissADME, a free-to-access web tool (http://www.swissadme.ch/) for instant and robust prediction of physicochemical properties and pharmacokinetic descriptors including ADME parameters. The SwissADME tool utilizes the most relevant computational methods for efficient calculation of Drug likeness (DL), Oral Bioavailability (OB), Caco-2 permeability, lipophilicity, and other features of naturally occurring small molecules. The estimation of the drug-likeness of the compound was calculated by Lipinski and other rules (Daina et al., 2017).

#### 2.1.2 Identification of 18α-GA-related targets

The chemical structure of 18α-GA as an SDF file was obtained from the PubChem database (https://pubchem.ncbi.nlm.nih.gov/). The structure of 18α-GA was imported into PharmMapper (http://lilab.ecust.edu.cn/pharmmapper/) (Wang et al., 2017) and SwissTarget (Daina et al., 2019) database for identifying related target genes. The list of putative target genes was downloaded as a CSV file and the gene names were changed to standard gene symbols utilizing the UniProt database (http://www.uniprot.org/).

#### 2.1.3 Collection of gene targets specific to Non-small cell lung cancer

The pathogenic gene targets of NSCLC were extracted from DisGeNET (version 6.0) database after setting the species as homosapiens. DisGeNET is the largest platform linking human genes to diseases by integrating data from scientific literature, expert-curated repositories, and the genome-wide association study (GWAS) catalogs.

All overlapping gene targets shared by 18α-GA and NSCLC simultaneously were obtained by Venny 2.1.0 (http://bioinfo.cnb.csic.es/tools/venny/index.html) software and chosen as the expected target of 18α-GA compound for the treatment of NSCLC.

#### 2.1.4. Protein-protein interaction and network construction

The common target genes of 18α-GA and NSCLC were used to analyze the underlying mechanism of interacting genes and the protein-protein interaction network was constructed by importing the data to the STRING (Search Tool for the Retrieval of Interacting Genes 11.0) database (https://string-db.org/) (Szklarczyk et al., 2019). The species was specified as “*Homo sapiens*” and the cut-off confidence score of ≥0.9 was considered significant to assure robustness of the interaction. Further, the PPI network was visualized using the Cytoscape (version 3.8.2) (http://www.cytoscape.org/) software, and a Compound-Target- Disease (“C-T-D”) network was constructed (Shannon et al., 2003). The network nodes represent proteins whereas the edge represents associated protein-protein interaction.

#### 2.1.5 Prediction and validation of hub/core targets

The CytoHubba plugin of Cytoscape was utilized to analyze the highly interconnected nodes in the PPI network that represents the most important interacting genes of a network. The top overlapping genes among important topological features like Degree, Betweenness, Closeness, and Maximal Clique Centrality (MCC) were considered to filter core genes in the network.

#### 2.1.6 Molecular docking

Next, the molecular docking approach was applied to confirm the interaction strength between the compound 18α-GA and the predicted core targets. The 3D structures of core proteins were obtained from the RCSB PDB database (https://www.rcsb.org/) and the structure of the compound was taken from the PubChem database and converted to PDB format through Pymol software (version 2.2). During the receptor preparation module, a PDBQT file of our target receptors was generated that consisted of atoms of hydrogen along with partial charges. There are multiple ways to estimate partial charges but we used “Kollman charges” which provide a quick and simple calculation of receptor topology. Furthermore, we added hydrogen atoms to the PDB receptor files as well as deleted water molecules and converted them into the PDBQT format for docking calculations. In proteins, the active site has been predicted through the CASTp 3.0 (Computed Atlas of Surface Topography) online server (Tian et al., 2018). CASTp is a good platform to predict and characterize the active site based on algorithmic computational geometry and identify the pocket and cavities analytically. Additionally, the grid was made around the active site (catalytic pocket). InstaDock has been used for molecular docking calculation (Mohammad et al., 2021) and the results were evaluated from log files generated by docking performance. After molecular docking, the docked conformations were analyzed by Discovery Studio 2019 for 3D and 2D interaction analysis.

#### 2.1.7 Functional enrichment analysis

The GO and KEGG signaling pathways enrichment analyses of screened gene targets were done by entering targets of 18α-GA in the DAVID database (Huang et al., 2007). The Gene Ontology terms like biological process (BP), cellular component (CC), and molecular function (MF) were analyzed and the results with values of *p* < 0.05 and FDR <0.05 were considered to be statistically significant. The Top 15 terms were plotted into graphs using GraphPad.

#### 2.1.8 Survival analysis

The association between the survival rates of NSCLC patients and the expression levels of the core targets was analyzed using the Kaplan-Meier Plotter (https://kmplot.com) (Lánczky and Győrffy, 2021). Furthermore, the Gene Expression Profiling Interactive Analysis (GEPIA2) webserver (http://gepia2.cancer-pku.cn) was used for performing tumor/normal differential core gene expression analysis.

Besides, the Human Protein Atlas (http://www.proteinatlas.org) database was used to observe the distribution of hub proteins in the lung cancer cells and normal lung cells.

### 2.2 Experimental verification

#### 2.2.1 Chemicals and reagents

High-glucose Dulbecco’s Modified Eagles Medium (DMEM), Fetal Bovine Serum (FBS), and the penicillin-streptomycin mixed solution were procured from Gibco-life technologies, Thermo Fischer Scientific (USA). MTT (3-[4,5-dimethylthiazol-2-yl]-2,5-diphenyltetrazolium bromide), propidium iodide (PI), DAPI, Acridine orange were acquired from Sigma. Bax, *β*-actin, and Bcl-2 primary antibodies were purchased from Novus Biologicals (Centennial, CO, USA). FITC/Annexin V apoptosis detection kit was purchased from BD Bioscience. The compound 18α-GA was purchased from Sigma-Aldrich with purity ≥95% and stock solutions were dissolved in DMSO and stored at −20°C. All other reagents were of analytical grade and purchased from local suppliers. All the experiments were repeated at least three times.

#### 2.2.2 Cell culture

The NSCLC cell lines H1299 and A549 were procured from National Centre for Cell Sciences (NCSS), Pune, India. All the cell lines were cultured in DMEM, supplemented with 1% 1X penicillin/streptomycin and 10% heat-inactivated FBS at 37°C and 5% CO_2_ under 95% humidified conditions in a cell culture incubator (Shell labs) (Alam et al., 2022). Cell cultures were maintained regularly, not more than 30 passages. The compound 18α-GA was dissolved in DMSO with a concentration not more than 0.2% and used as a vehicle in all the experiments.

##### 2.2.3 Cell Cytotoxicity assay

Cytotoxic potential of 18α-GA on NSCLC cell lines; H1299 and A549 were evaluated by MTT assay. Briefly, 6,000–7,000 cells were seeded per well in a 96-well plate and were allowed to grow overnight, the cells were then treated with increasing concentrations (0–200 µm) of 18α-GA for 24 and 48 h at 37°C in a CO_2_ incubator. After treatment, 10 µl MTT was added (5 mg/ml, PBS, pH 7.4) to each well, and incubated for 4–5 h at 37°C in a CO_2_ incubator in dark. The supernatant from each well was then discarded and 100 µl of DMSO was added. The absorbance was measured using a microplate reader (BioRad) at 570 nm. The IC_50_ values were calculated by GraphPad Prism 9.0 and percent cell inhibition was plotted for H1299 and A549 cells.

#### 2.2.4 Observation of morphological changes

H1299 and A549 cells were seeded 18α-GA at a density of 4 × 10^5^ cells per well in a 6-well culture plate and allowed to grow up to 70% confluency. Later, cells were treated with distinct concentrations of (0, 40, 80 µM) and incubated for 48 h. The cell morphology was observed and captured by using an inverted microscope (LMI microscopes, United Kingdom).

##### 2.2.4.1 DAPI staining

As previously described (Sun et al., 2022), H1299 and A549 cells were seeded at the density of 1×10^5^ cells/well in a 6-well plate overnight and treated with different doses of 18-αGA. After 48 h, the treated cells were washed with 1X PBS, followed by fixing using 3.7% paraformaldehyde for at least 10 min at RT. The fixed cells were further washed with PBS, stained by DAPI (300 nM)) and imaged under a fluorescence microscope (Bio-Rad).

#### 2.2.5 Colony formation assay

Briefly, 200–300 cells per well were grown overnight and treated with different doses of 18-αGA and allowed to form colonies in a serum-deprived medium (2%) for 10–14 days in a six-well cell culture plate. Subsequently, colonies were fixed (acetic acid/methanol; 1:3) and stained with 0.5% crystal violet in 100% methanol for 30 min, and then counted.

#### 2.2.6 Cell cycle assessment

Cells were treated as described above and then harvested, washed twice with 1×PBS, and fixed with 70% ethanol at 4°C for 24 h. Next, cells were washed with PBS and incubated with PI staining solution (50 μg/ml PI and 100 µg/ml RNAase A) for 30–40 min at 37°C in dark. The percentage of cell count in the G0, G1, S, and G2/M phases of the cell cycle was determined by BD FACS Aria BD FACS Aria™ III (BD Biosciences, United States). A total of 10,000 events for all samples were taken to ensure adequate data.

#### 2.2.7 Acridine orange/ethidium bromide staining

In addition to DAPI staining, acridine orange (AO) and ethidium bromide (EB) staining were carried out to detect apoptotic cell formation. The assay was performed as described previously with slight modifications (Kasibhatla et al., 2006). Briefly, cells were treated as described above and then spent media was collected, cells harvested, and centrifuged. After washing with PBS, 25 µl of cell suspension was stained with 1 µl of AO/EB solution (1:1) and incubated for 1–2 min. Subsequently, fluorescence images were captured under a fluorescence microscope (Bio-Rad).

#### 2.2.8 FITC annexin V/propidium iodide apoptosis assay

FITC Annexin V Detection Kit I (BD-Biosciences, USA) was used to measure the apoptotic changes in both the cell lines according to manufacturers’ instructions. Briefly, 1 × 10^6^ cells/mL were seeded in a 6-well plate and treated with different concentrations of 18α-GA for 48 h. After harvesting, the cells were suspended in 500 µl of binding buffer. 100 µl of this cell suspension were mixed with 5 ml of Annexin V and propidium iodide (PI) for 15–20 min at RT in dark. The percentage of live and dead cells was determined using FITC/PI channel, and 10,000 events for each sample were recorded by BD FACS Aria™ III (BD Biosciences, USA) using BD FACS Diva software.

#### 2.2.9 Mitochondrial membrane potential measurement

We assessed the mitochondrial transmembrane potential using the Tetramethylrhodamine, methyl ester (TMRM, Invitrogen) fluorescent dye (Creed and McKenzie, 2019). After being treated with different concentrations, H1299 and A549 cells were incubated with 200 nM TMRM in DMEM for 30 min at 37°C in dark. After washing three times with PBS, the cell monolayer was imaged under a fluorescence microscope.

#### 2.2.10 Wound healing assay

The assay was performed as previously described with minor changes. In brief, 1 × 10^5^ cells were allowed to form a monolayer in a 12-well plate, then a scratch was made using a 10 μl pipette tip. After thorough and gentle washing, the cells were treated accordingly under serum-starved conditions for different time -points. Images were obtained at 0, 24, and 48 h to analyze the movement of the cells to close the wound. ImageJ software (National Institutes of Health, Bethesda, MD, United States) was used to analyze the migration distance.

#### 2.2.11 Semi-quantitative RT-PCR

Total RNA was extracted from treated cells using Trizol reagent (Invitrogen). Next, cDNA was transcribed using an iScript cDNA synthesis kit (Bio-Rad) according to the manufacturer’s protocol. RT-PCR analyses for mRNA of p53, p27, cyclin E1, E-cadherin, vimentin, and snail, were performed by using the GoTaq Green PCR master mix (Promega). GAPDH was used as the endogenous control for data normalization. The PCR amplicons were run on 1.8% agarose gel and visualized on the ChemiDoc imaging system (Bio-Rad) and the bands were quantified using ImageJ (Bethesda, Maryland, MD, United States) software.

#### 2.2.12 Western blot

The experiment was performed as described previously (Padder et al., 2021). Briefly, H1299 and A549 cells were treated with different concentrations of 18α-GA for 48 h, trypsinized, and lysed in RIPA lysis buffer supplemented with 1x Phos stop and Sigma fast (Sigma Aldrich) for 10–15 min on ice. The lysates were centrifuged at 10,000 rpm for 10 min at 4°C; the supernatant was taken and total protein was quantified using a BCA kit (Thermo Scientific). Equal amounts of protein (20–50 μg) were resolved on SDS-PAGE gels, followed by transfer of the resolved proteins onto PVDF membrane (Bio-Rad) using a wet transfer system at 60 V–80 V for 1 h. The membranes were then blocked with 5% non-fat milk in TBST for 40 min and probed overnight with indicated primary antibodies at 4°C and later probed with appropriate HRP conjugated secondary antibodies at room temperature for 1 h. The protein bands were then developed onto x-ray film (kodak) and the ChemiDoc imaging system. The primary antibodies used were against, beta-actin, caspase-3, Bax, and Bcl_2_ (Novus Biologicals), EGFR and pPKBa (Cloud Clone), pPI3K (CST), and the corresponding secondary antibodies used were either goat anti-rabbit IgGHRP (Novus Biologicals) or goat anti-mouse IgGHRP, (Novus Biologicals).

#### 2.2.13 Statistical analysis

Data analysis was performed using Graph Pad prism 9.0. All experiments were repeated at least three times independently. Data are presented as the mean ± SD. Differences between groups were analyzed by two-way ANOVA as applicable with appropriate post hoc tests as indicated. *p* values corresponding to *p* ≤ 0.05 were considered statistically significant.

## 3 Results

### 3.1 Bioinformatic study

#### 3.1.1 Putative targets of 18α-GA against non-small cell lung cancer

**FIGURE 1:**
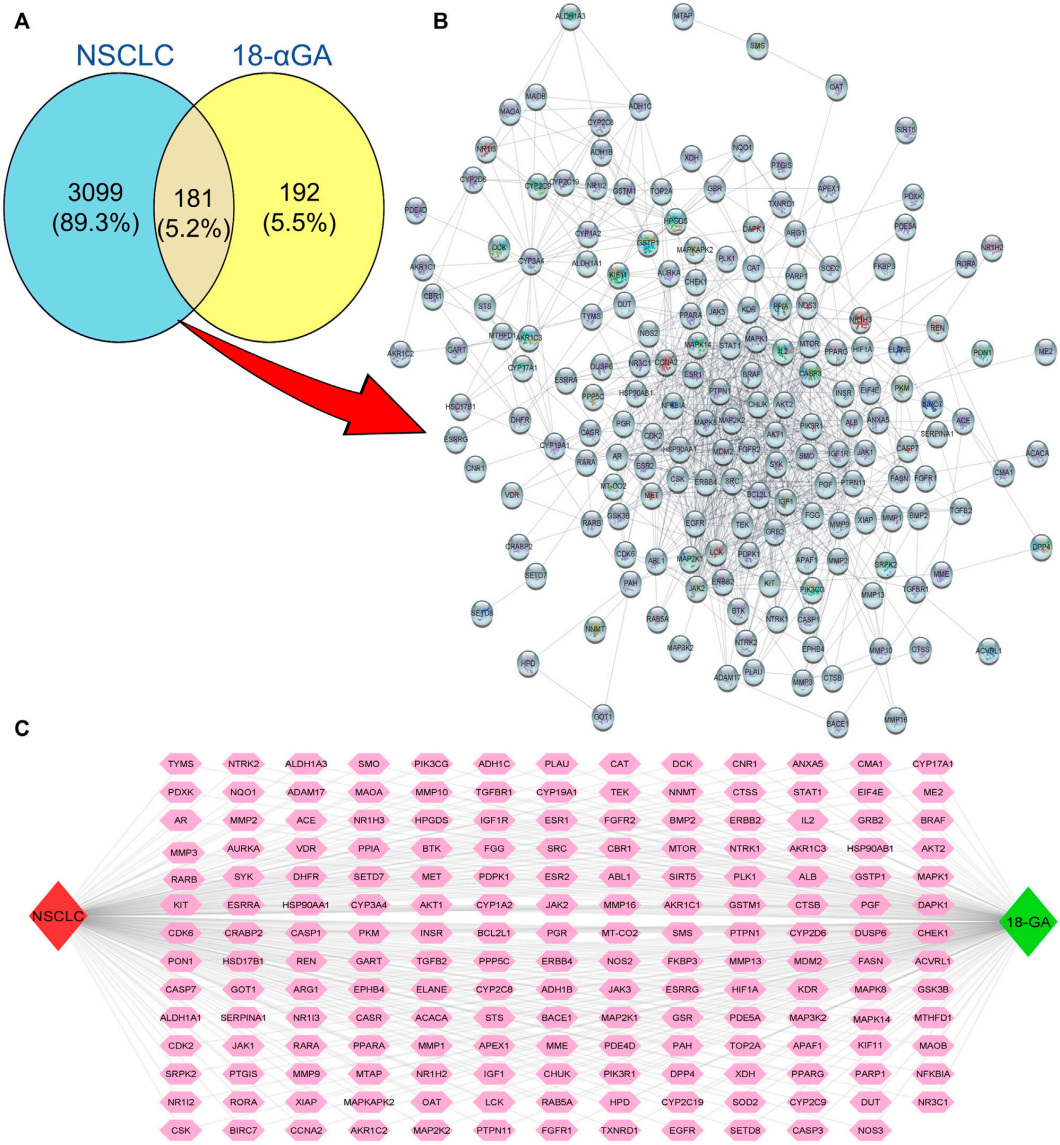
Putative targets of 18-αGA against NSCLC. **(A)** Venn Diagram showing 181 overlapping targets between 18-αGA and NSCLC by Venny 2.1.0. **(B)** Protein-protein interaction (PPI) network of 181 nodes and 964 edges of 18-αGA and NSCLC as visualised by *Cytoscape v 3.9.0*. **(C)** The “Compound-Target-Disease” Network constructed by Cytoscape v.3.8.2, the Green diamond represents the compound name, the Red diamond represents the disease name, and Pink hexagons show common targets between 18-αGA and NSCLC.

Natural products have the characteristic ability to act on multiple molecular targets and hence can be utilized to harness multi-targeted therapeutic strategies against various diseases. The PharmMapper and SwissTarget online tools are online databases that match the query compound against a huge internal pharmacophore model database for potential drug target identification *via* a reverse pharmacophore alignment. Thus, 305 and 100 putative targets associated with 18α-GA were identified using PharmMapper and SwissTarget, respectively. Later, the DisGeNET database predicted a total of 3926 NSCLC-related genes, which, when overlapped using a Venn diagram, produced 181 potential targets of 18α-GA against NSCLC (Figure 1A). These predicted targets were subjected to further exploration.

#### 3.1.2 “C-T-D network” construction and hub/core targets identification

**FIGURE 2:**
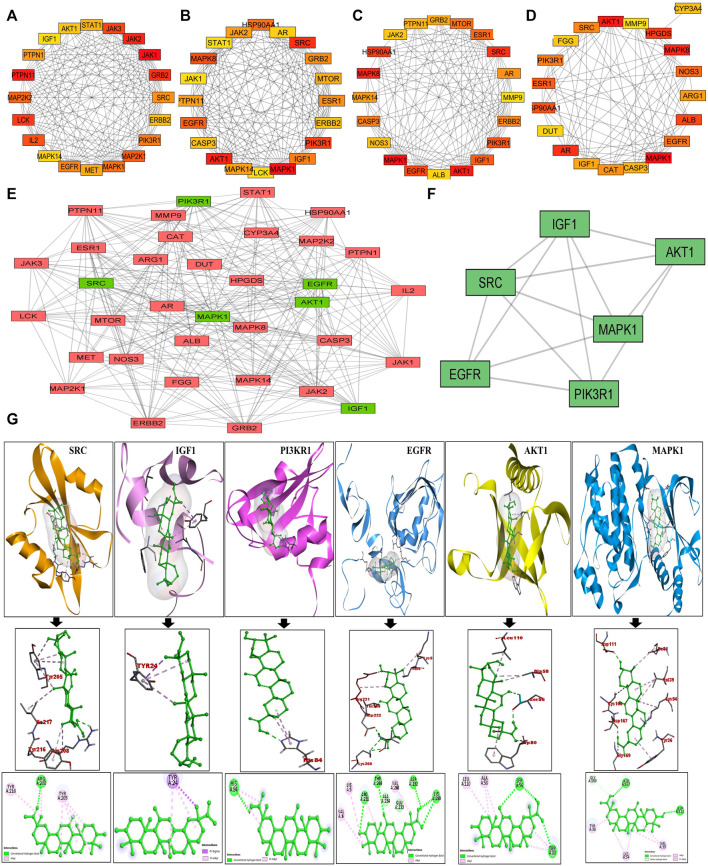
Identification of Top 6 hub/core targets of 18-αGA against NSCLC. **(A)** Top 20 targets based on MCC visualized by CytoHubba plugin. **(B)** Top 20 targets based on Degree as visualized by CytoHubba plugin. **(C)** Top 20 targets based on Betweenness as visualized by the CytoHubba plugin **(D)** Top 20 targets based on Closeness as visualized by the CytoHubba plugin. **(E)** The PPI network of identified hub/core targets of 18-αGA against NSCLC constructed by merging top 20 targets obtained from CytoHubba plugin visualized by Cytoscape v 3.8.2; green rectangles represent the highly enriched target nodes and pink boxes show less enriched nodes. **(F)** Top 6 hub nodes interactive PPI network visualized and constructed by Cytoscape v3.8.2.**(G)** Molecular docking verification of the crucial hub targets of 18-αGA against NSCLC. The three-dimensional structure of 18-αGA docked against the structure of the targets is shown along with the interaction diagram. The arrow represents the docking complexes in 3D and 2D poses respectively.

The putative targets obtained were imported to the STRING platform and a PPI network was retrieved (Supplementary Figure S2) and visualised by *Cytoscape v3.9.0* (Figure 1B). The PPI network reveals the interaction among the targets with more interacting lines between proteins representing highly connected proteins in the network. A“Compound-Target-Disease” network was constructed using the *Cytoscape 3.8.2* software (Figure 1C). The resultant “C-T-D” network contained 181 nodes and 964 edges representing proteins and the interaction among them respectively. The red and green diamonds represent NSCLC and 18α-GA respectively, whereas the pink hexagons represent the putative protein targets of 18α-GA acting against NSCLC. To further understand the above network comprehensively, topological analysis was done by a Cytoscape plugin, Cytohubba, which exhibited the top 20 proteins related to degree, betweenness centrality, closeness centrality, and maximum clique centrality as shown in (Figures 2A–D). Furthermore, these top-rated proteins were merged and intersected revealing the 6 most important and highly connected proteins the Merge tool of Cytoscape 3.8.2. Figure 2E represents the merged PPI network with green boxes illustrating the most crucial proteins in the network that signifies the hub/core targets of 18α-GA effective against NSCLC. The 6 core hub proteins identified as therapeutic targets were; EGFR, IGF1, MAPK1, AKT1, PIK3R1, and SRC (Figure 2F).

#### 3.1.3 Molecular docking of core targets

These hub proteins were docked against 18α-GA to understand the binding interactions among them. When subjected to molecular docking analysis, the binding energies of hub targets from PPI network analysis demonstrated high affinities towards 18α-GA with EGFR, MAPK1, and AKT1 having the lowest binding energies of -8.8 kcal/mol, −8.2 kcal/mol, and −7.9 kcal/mol respectively and the number of interacted bonds (polar and non-polar) showed the ligand is stable in the binding pocket (Supplementary Table S2). The docking conformations of hub targets are shown in Figure 2G, highlighting the stable ligand-receptor binding and greater interaction. Most of the docked targets with high binding affinities were distributed in crucial pathways revealed from GO and KEGG analysis, hence validating the role of ErbB and PI3K signaling pathways.

#### 3.1.4 Functional enrichment analysis

**FIGURE 3:**
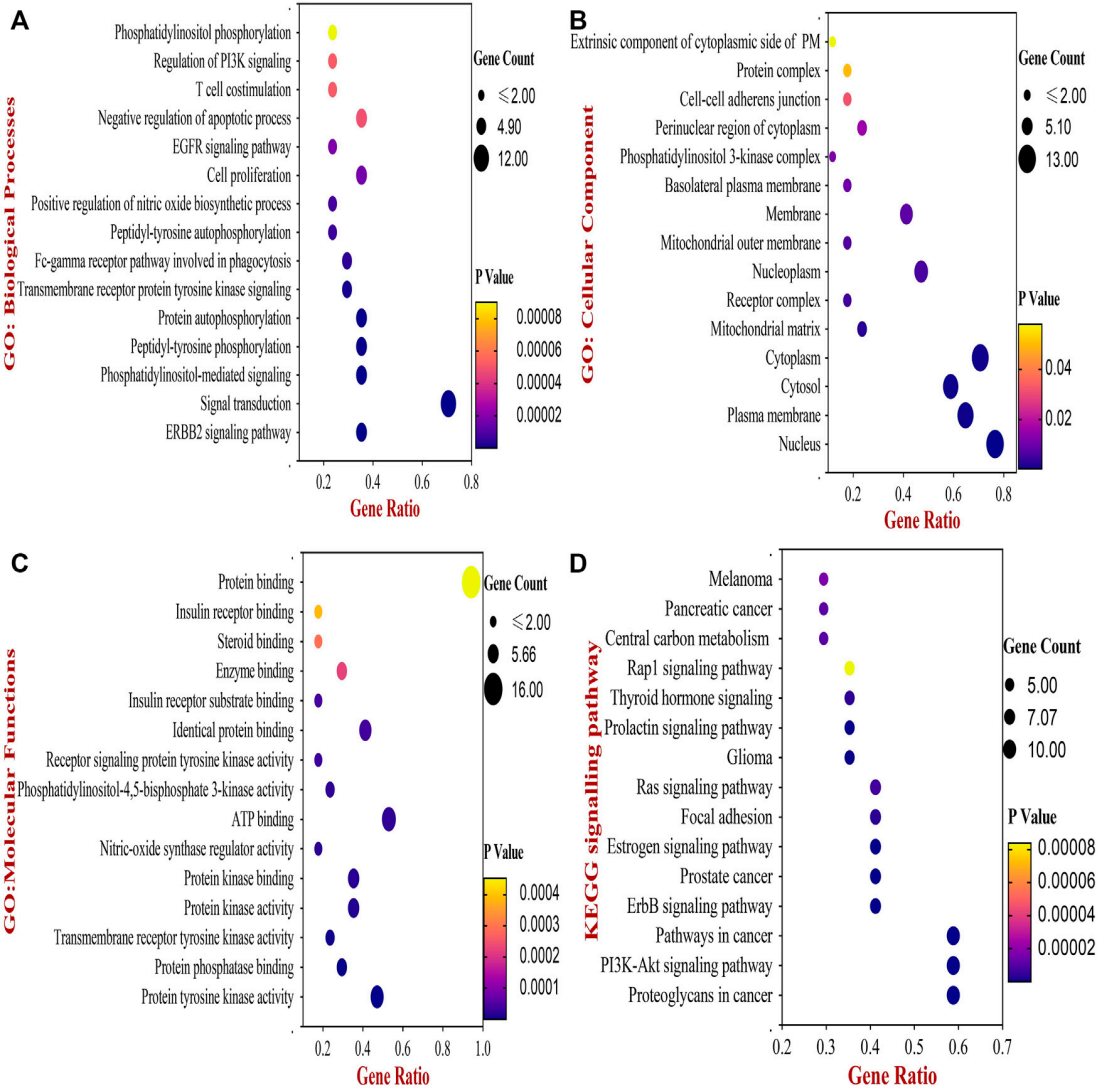
The Bubble chart of Gene Ontology (GO) and KEGG pathway analyses by DAVID database. **(A)** Top 15 GO- Biological Processes of 18-αGA targets. **(B)** Top 15 GO- Cellular Component of 18-αGA targets. **(C)** Top 15 GO- Molecular Functions; *p*-value < 0.05 in all three GO terms **(D)** Bubble plot of Top 15 highly enriched KEGG pathways of 18- αGA targets, *p*-value < 0.05. Bubble size represents the number of enriched genes, and bubble color difference represents the significance of target gene enrichment.

Next, the biological functions and pathways of anti- NSCLC targets of 18α-GA were elucidated by enriching KEGG pathways and acquiring GO terms by DAVID software. After correcting the *p*-values, the top 20 most significant GO terms associated with Biological Processes (GO_BP), Cellular components (GO_CC), and Molecular Functions (GO_MF) were visualized in Figures 3A–C. According to the GO analysis, the most vital biological processes involved in the anti-NSCLC activity of 18α-GA were attributed to the ERBB2 signaling pathway, signal transduction, PI-mediated signaling, cell proliferation, negative regulation of apoptosis, regulation of phosphatidylinositol 3-kinase (PI3K) mediated signaling and peptidyl-tyrosine phosphorylation among others. The most valued GO_CC terms involved, were the nucleus, cytoplasm, plasma membrane, cytosol, and nucleoplasm. In GO_MF, the most important terms included were protein tyrosine kinase activity, protein phosphatase binding, identical protein binding, transmembrane receptor protein tyrosine kinase activity, and phosphatidylinositol-4,5-bisphosphate 3-kinase activity among others.

Following this, enrichment of the KEGG pathway disclosed a total of 56 pathways involved in the anti- NSCLC effect of 18-αGA. After adjustment of the *p*-value, the top 15 pathways encompassed proteoglycans in cancer, PI3K-Akt signaling pathway, ErbB signaling pathway, Pathways in cancer, Estrogen signaling pathway, Focal adhesion, Ras signaling pathway, and Prolactin signaling pathway (Figure 3D). The integration of the above GO and KEGG data indicates that the 6 hub targets are mainly enriched in the ErbB signaling pathway and PI3K-Akt signaling pathway, thereby associating significantly with the anti-lung cancer activity of 18-αGA. To this conjecture, we sought to perform further external validation of the hub/core targets by molecular docking, expression, and survival analysis.

#### 3.1.5 External validation by mRNA and protein expression of core targets

To validate the prognostic and clinical significance of the core targets, we used the GEPIA database to examine the differential expression of mRNA levels between NSCLC and normal lung tissues. The red and grey boxplots represent the lung cancer tissues and normal lung tissues respectively. It was observed that the mRNA expression of EGFR, MAPK1, Akt1, and SRC were upregulated in the lung cancer tissues whereas the expression of IGF1 and PI3KR1 was reduced as compared to the normal lung tissues (Supplementary Figure S3A).

Furthermore, protein expression levels of hub/core proteins by immunohistochemical staining images were studied from the HPA database. As shown in Supplementary Figure S3B, the staining indicating expression levels were moderate to strong in all hub proteins except for IGF1, which was undetectable in lung cancer tissues compared to normal lung tissue.

These assessments suggested that the expression levels of these hub genes/proteins are possibly correlated with NSCLC progression and could prove as potential biomarkers clinically for the detection and treatment of NSCLC.

#### 3.1.6 Survival analysis and prognostic value of the hub genes

Subsequently, to validate the clinical significance of targeting the hub genes, the association of NSCLC patients’ survival outcomes was co-related with the expression levels of these hub targets. The Kaplan-Meier plot (Supplementary Figure S3C) shows that high expression of EGFR, AKT1, MAPK, IGF1, and SRC significantly worsened the overall survival of patients while higher expression of PIK3R1 did not reduce the survival probability.

Collectively, the identification of hub targets of 18α-GA against NSCLC by network analysis and subsequent KEGG, GO, molecular docking, external expression analysis, and survival analysis verify the importance of particularly EGFR and AKT1 as the core therapeutic targets of 18α-GA among other hub genes. These findings confirm the prognostic and clinical significance of targeting these genes for restraining the pathogenesis of NSCLC by 18-αGA.

Taking into consideration these bioinformatics conclusions, further *in-vitro* experiments were performed to validate 18α-GA as an anti- NSCLC agent by modulating different hallmarks of cancer in a multi-targeted and multi-step manner. Therefore, it may be speculated that EGFR/AKT signaling pathway may be associated with the therapeutic mechanism of 18α-GA against NSCLC.

### 3.2 *In-vitro* verification-

#### 3.2.1 18α-GA inhibits cell proliferation in non-small cell lung cancer cells

**FIGURE 4:**
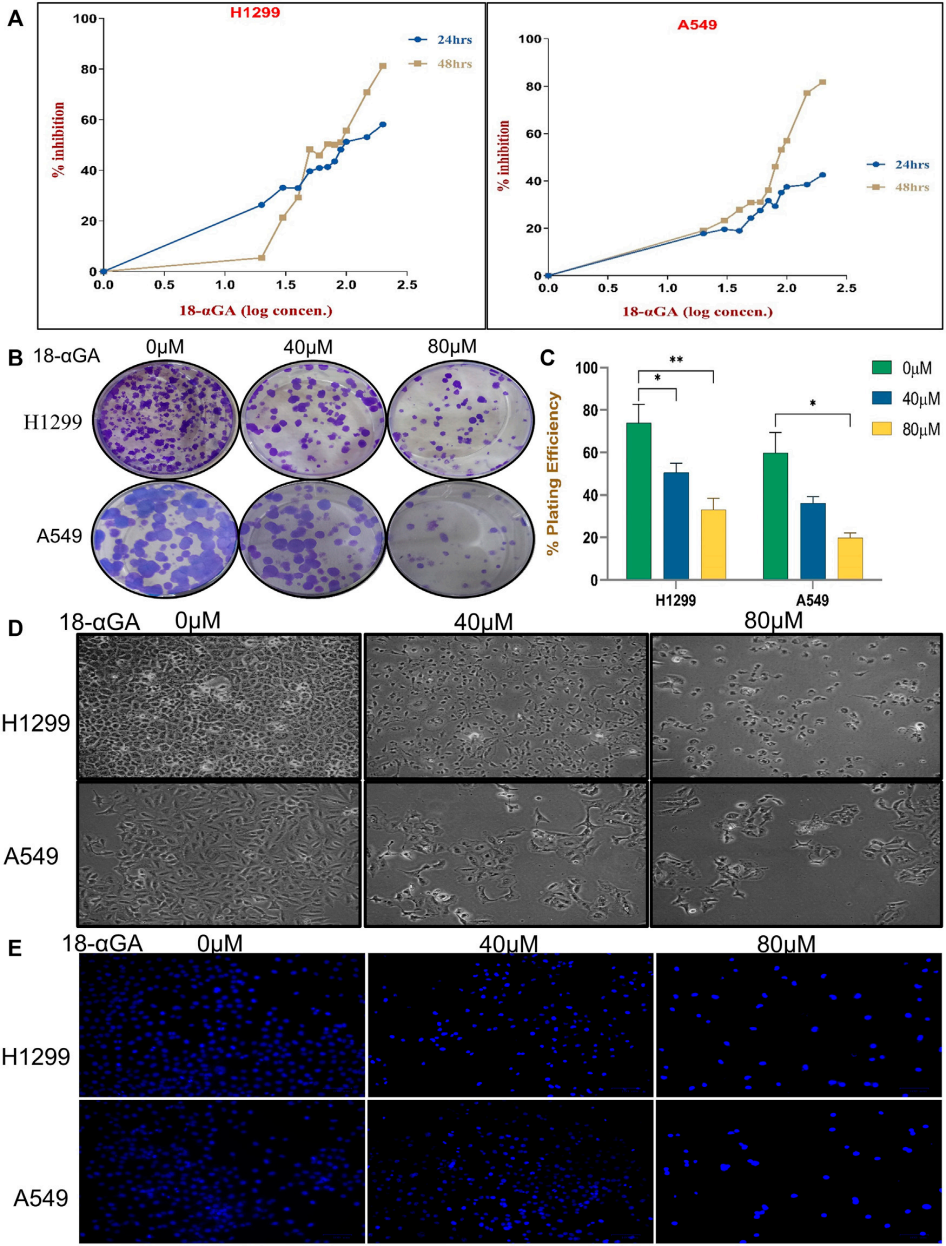
**(A)** Effect of 18-αGA on the viability and proliferation of NSCLC cells. **(A)** Growth inhibition assessment by the MTT assay upon treatment with various concentrations (0, 10, 20, 30,40, 50, 60, 70,80, 90, 100, 150, and 200 μM) of 18-αGA at two different time intervals of 24 h and 48 h on NSCLC cell lines; H1299 and A549. **(B)** Images showing colonies appeared after treatment at two different concentrations in both cell lines. **(C)** % plating efficiency after treatment with 18-αGA, colonies counted by ImageJ (*n* = 3, mean ± SD), statistical analysis was done by 2-way Anova followed by Tukey’s Post-hoc multiple comparison test. *p < 0.05, **p < 0.01, ***p < 0.001, ****p < 0.0001. **(D)** Cell morphological and cytoplasmic changes in H1299 and A549 cells in the different treatment groups after 48 h **(E)** DAPI staining shows changes in the fluorescence levels of cells in different 18-αGA treatment groups (original magnification, × 20).

To prove the data obtained through bioinformatics, further study was done by performing experiments in cell culture. First, the growth-suppressive effects of 18α-GA were assessed with different concentrations of 18α-GA at 2 different time points (24 h and 48 h). At 24 h, the MTT assay revealed that in H1299 cells, the inhibition was prominent whereas, in the A549 cells, the viability was not significantly affected up to 200 µM. Besides, H1299 and A549 cell proliferation were significantly inhibited at 48 h in a dose and time-dependent manner with more than 80% inhibition at higher concentrations (Figure 4A). The IC50 values as calculated by Microsoft Excel and were found to be 111.17 µM and >200 µM for H1299 and A549 cells respectively at 24 h. Similarly, at 48 h, the IC50 values were found to be 73.45 µM and 91.20 µM and it was noted that H1299 cells were more responsive to 18α-GA than A549 cells at both time points. Accordingly, 40 µM and 80 µM treatments for 48 h were chosen to carry out further experiments in both the cell lines.

The cell viability of non-cancer cell line HEK-293 at different concentrations of 18α-GA at 24 and 48 h was also measured, which showed negligible effect and the cells were viable even after 48 h of treatment at the highest dose of 18α-GA (Supplementary Figure S4).

Further, the long-term effect of 18α-GA on the proliferation of NSCLC cell lines was assessed by colony formation assay. A significant reduction in the number of colonies was observed with a concomitant decrease in the size of the cell colonies (more prominent in A549 cells) as compared to the untreated group, indicating the growth inhibitory potential of 18α-GA in both the cell lines with increasing concentrations (Figures 4B,C).

#### 3.2.2 18α-GA causes morphological changes in non-small cell lung cancer cells

For evaluating the effects of 18α-GA as predicted by bioinformatic, two NSCLC cell lines; H1299 and A549 were exposed to two different concentrations of 18α-GA, and the morphological changes in the cells were observed under an inverted microscope. After 48 h, a disrupted monolayer with cells floating in the media along with rounding, shrinkage, and loss of characteristic size and shape of the cells was observed as shown in Figure 4D. To further evaluate these changes, DAPI staining was done. It revealed that with increasing concentration, cells became more and more sparse with a decrease in the cell numbers and density. Although, a significant increase in blue fluorescence of the treated cells was observed clearly indicating permeabilization of the membrane marking cell death (Figure 4E).

#### 3.2.3 Cell cycle progression was arrested at G0/G1 stage

**FIGURE 5:**
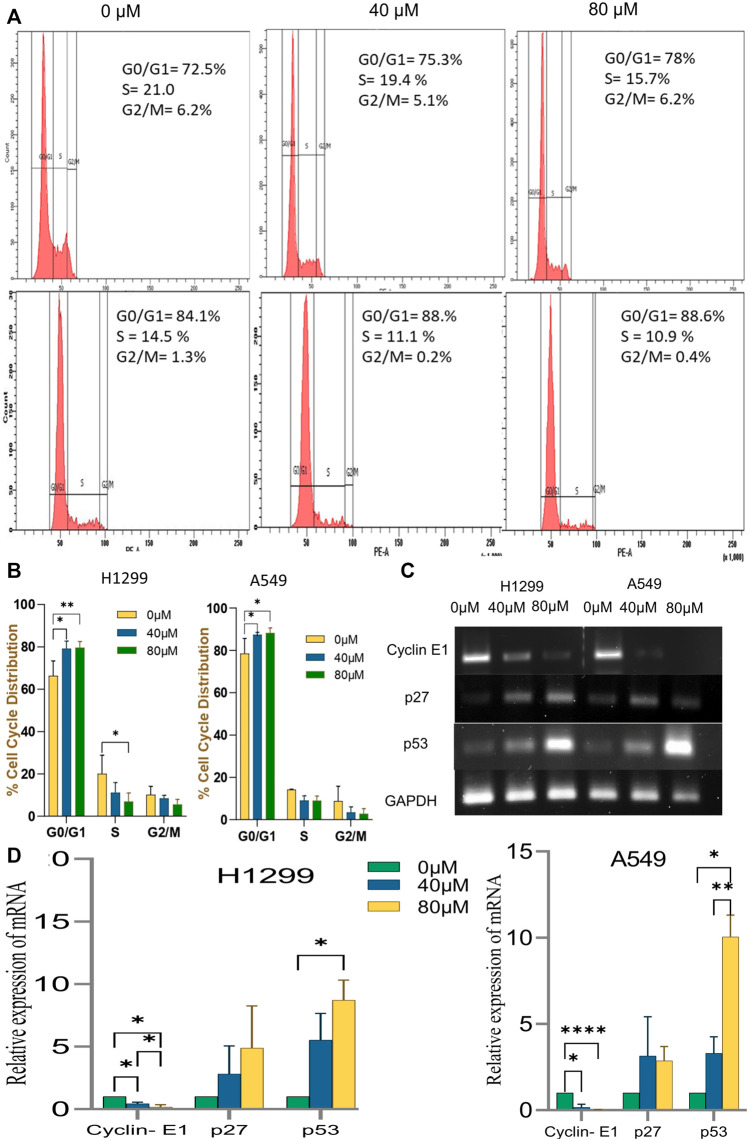
Effect of Cell Cycle progression after treatment with 18α-GA. **(A)** Flow cytometric images represent the distribution of H1299 and A549 cells at different stages of the cell cycle. **(B)** % cell cycle distribution measured by FACS (*n* = 3, mean ± SD), statistical analysis was done by 2-way Anova followed by Tukey’s Post-hoc multiple comparison test. *p < 0.05, **p < 0.01, ***p < 0.001, ****p < 0.0001. **(C)** The mRNA expression of related genes after treatment with 18α-GA by RT-PCR. **(D)** Graph representing mRNA expression levels of genes with respect to GAPDH, statistical analysis was done by 2-way ANOVA followed by Tukey’s Post-hoc multiple comparison test, *p < 0.05, **p < 0.01, ***p < 0.001, ****p < 0.0001.

Disruption of the cell cycle process is a hallmark of cancer cells. After treatment with 18-αGA, cell cycle analysis by flow cytometry revealed that the cells remained significantly at the G1 stage at both concentrations in H1299 and A549 cells (Figures 5A,B). This increase in cell number suggests that the cell cycle was arrested at the G1 phase. To know the possible molecular mechanisms implicated with growth arrest in NSCLC cells, regulatory proteins of the cell cycle were examined by RT-PCR. The cell cycle is a complex mechanism regulated by the coordination of several markers and the mRNA expression analysis of G1/S transition markers Cyclin E1, p27, and p53 were analyzed (Figures 5C,D). It was observed that the mRNA expression of p53 and p27 were increased significantly in the treated cells whereas the expression of the cyclin E1 gene was decreased significantly in a representing cell cycle arrest at the G1 phase.

#### 3.2.4 18α-GA induces apoptosis in non-small cell lung cancer cells

**FIGURE 6:**
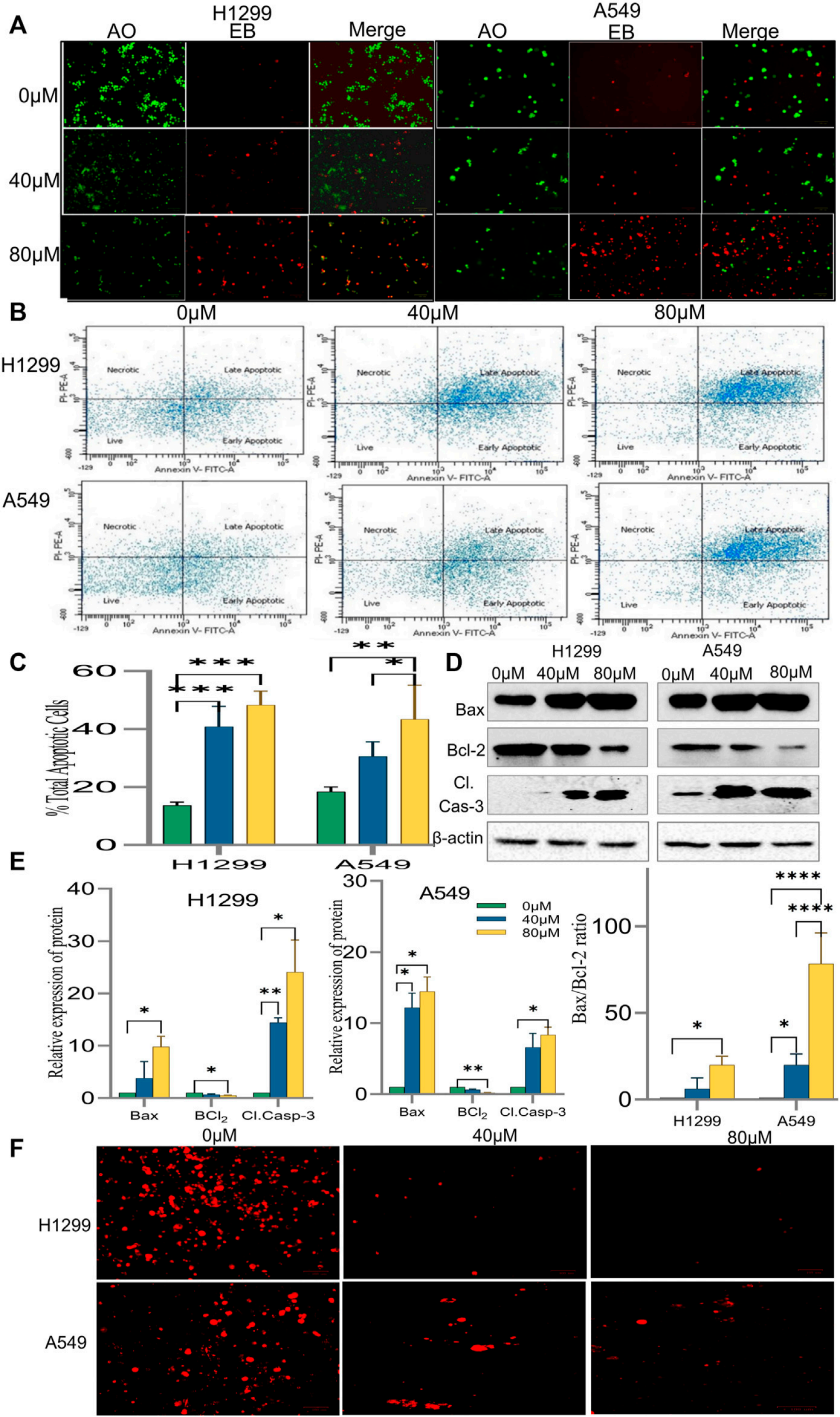
Induction of apoptosis by 18α-GA in H1299 and A549 cells. **(A)** Images of AO/EB staining by fluorescence microscopy; Green fluorescence represents healthy cells, Red cells represents cells undergoing late apoptotic stage. **(B)** Fluorescent images showing cell distribution in different stages of apoptosis detected by FACS. **(C)** % total apoptotic cells found after treatment with 18-αGA; (*n* = 3, mean ± SD), statistical analysis was done by 2-way ANOVA followed by Sidak’s Post-hoc multiple comparison test. *p < 0.05, **p < 0.01, ***p < 0.001, ****p < 0.0001. **(D)** Western blots showing expressions of apoptotic-related proteins. **(E)** Graphs representing protein expression levels after normalisation with beta-actin, statistical analysis was done by 2-way ANOVA followed by Tukey’s Post-hoc multiple comparison test, *p < 0.05, **p < 0.01, ***p < 0.001, ****p < 0.0001. Graph representing calculated Bax/Bcl-2 ratio, Sidak’s multiple comparison test was used. **(F)** Fluorescent images show TMRM staining for mitochondrial membrane potential.

It was speculated that the morphological changes and growth inhibition observed may be due to apoptotic induction in the cells. Accordingly, AO/EB fluorescent imaging revealed that the exposure of cells to 18α-GA caused in reduction of bright green fluorescence and an increase in red fluorescence indicating a decrease in healthy viable cells and an increase in dead late apoptotic cells respectively as compared to the control group. Additionally, orange-red fluorescence in the treated groups of H1299 cells was more prominent indicating that these cells were undergoing early apoptotic changes (Figure 6A).

The above results were substantiated by flow cytometric analysis after Annexin V/PI double staining. The FACS analysis exhibited a significant increase in the percentage of cells undergoing early and late apoptotic stages. It was 47.3% and 53.8% in treated H1299 cells in their respective concentration when compared to control cells, which was 21.2%. Similarly, in treated A549 cells, it was 34.2 and 53.8% in their respective concentration as compared to 19.8% in control cells. (Figures 6B,C). Also, a shift of cells from the early apoptotic stage to the late apoptotic stage was observed at higher concentrations in both H1299 and A549 cell lines indicating cell death.

To confirm the induction of apoptosis after exposure to 18-αGA, protein expression levels of apoptotic-related markers i.e., Caspase-3, Bcl2, and Bax were analyzed by western blotting.

The results in Figures 6D,E demonstrated that pro-apoptotic marker Bax and cleaved caspase-3 expressions were increased markedly in the treated groups as compared to the untreated one in H1299 cells. In A549 cells, the expression of these proteins was slightly less. Expectedly, the expression of anti-apoptotic protein Bcl_2_ was significantly diminished in both the cell lines at both concentrations as compared to the control group. A fine balance among Bcl-2 family proteins leads to apoptotic regulation in cancer cells, as noted above, the simultaneous increase and decrease of Bax and Bcl-2 proteins respectively led to an almost 70% increase in the A549 cel lines whereas the increase was nearly 20% in H1299 cells in the Bax/Bcl-2 ratio (Figure 6E) at 80 µM as compared to control cells validating the induction of apoptosis after exposure to 18-αGA (Zhu et al., 2015).

#### 3.2.5 18α-GA causes loss of mitochondrial membrane potential in non-small cell lung cancer cells

To understand the involvement of mitochondria in the apoptotic effect of 18α-GA on H1299 and A549 cells, mitochondrial membrane potential analysis was done. In early apoptotic cells, depolarization of the mitochondrial membrane occurs, and the MMP decreases. TMRM is a cell-permeant, cationic, and red-orange fluorescent dye readily sequestered by active mitochondria. In apoptotic cells, TMRM leaves mitochondria and dye dispersion occurs.

As presented in Figure 6F, it was observed that in both the cell lines, the treated cells have significantly decreased red fluorescence cells compared to untreated ones, indicating malfunctioning of the mitochondrial membrane potential. This decrease in the MMP and disruption of membrane potential gradient indicates that the 18α-GA induces apoptosis at both concentrations in both cell lines.

#### 3.2.6 18α-GA confers a reduction in the migratory potential of non-small cell lung cancer cells

**FIGURE 7:**
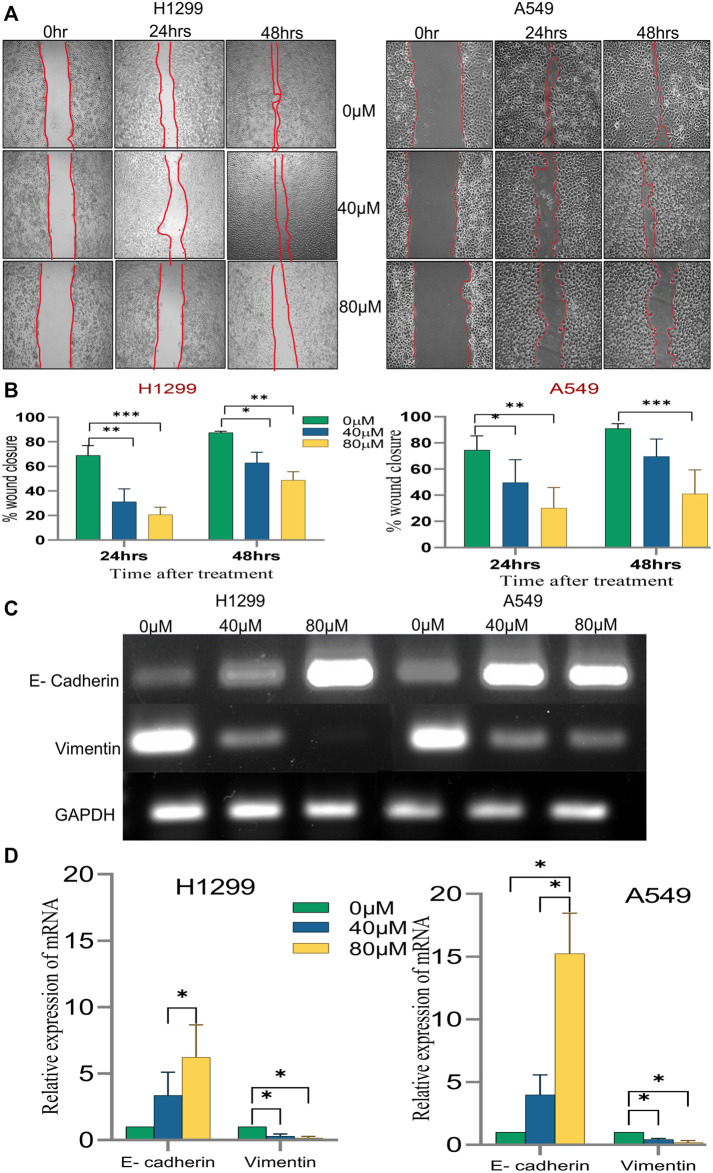
The effect of different concentrations of 18α-GA on the migration of NSCLC cells. **(A)** Images representing wound healing assay at two different time points, the wound area was calculated by ImageJ. **(B)** % wound closure calculated after treatment with 18α-GA (*n* = 3, mean ± SD), statistical analysis was done by 2-way ANOVA followed by Bonferroni’s Post-hoc multiple comparison test, *p < 0.05, **p < 0.01, ***p < 0.001, ****p < 0.0001 **(C)** The mRNA expression of related genes after treatment with 18α-GA by RT-PCR. **(D)** Graph representing mRNA expression levels of genes with respect to GAPDH, statistical analysis was done by 2-way ANOVA followed by Tukey’s Post-hoc multiple comparison test, *p < 0.05, **p < 0.01, ***p < 0.001, ****p < 0.0001.

Since the migratory potential of tumor cells is essential for metastatic dissemination and tumorigenesis, the effect of 18α-GA on H1299 and A549 cancer cells was evaluated for metastatic propensity. The results of the wound-healing assay (Figure 7A) exhibited a significant dose-dependent decrease in the wound closure ability of the cells in the treated groups compared to the untreated ones. The cell migration in the wound healing assay showed reduced migration of cells in the wound as compared to the control group at both concentrations in both the cell lines suggesting abrogation of the wound healing capacity of the cells after pre-treatment with 18-αGA (Figure 7B).

Next, it was sought to check the key EMT markers at the molecular level. It was found that the mRNA expression of E-cadherin was intensified significantly in the treated groups with a concomitant decrease in the vimentin mRNA expression (Figures 7C,D). These outcomes at least in part suggest that 18α-GA could significantly inhibit the EMT ability of H1299 and A549 cells.

#### 3.2.7 18α-GA inhibits EGFR-phosphatidylinositol 3-kinase/AKT pathway

**FIGURE 8:**
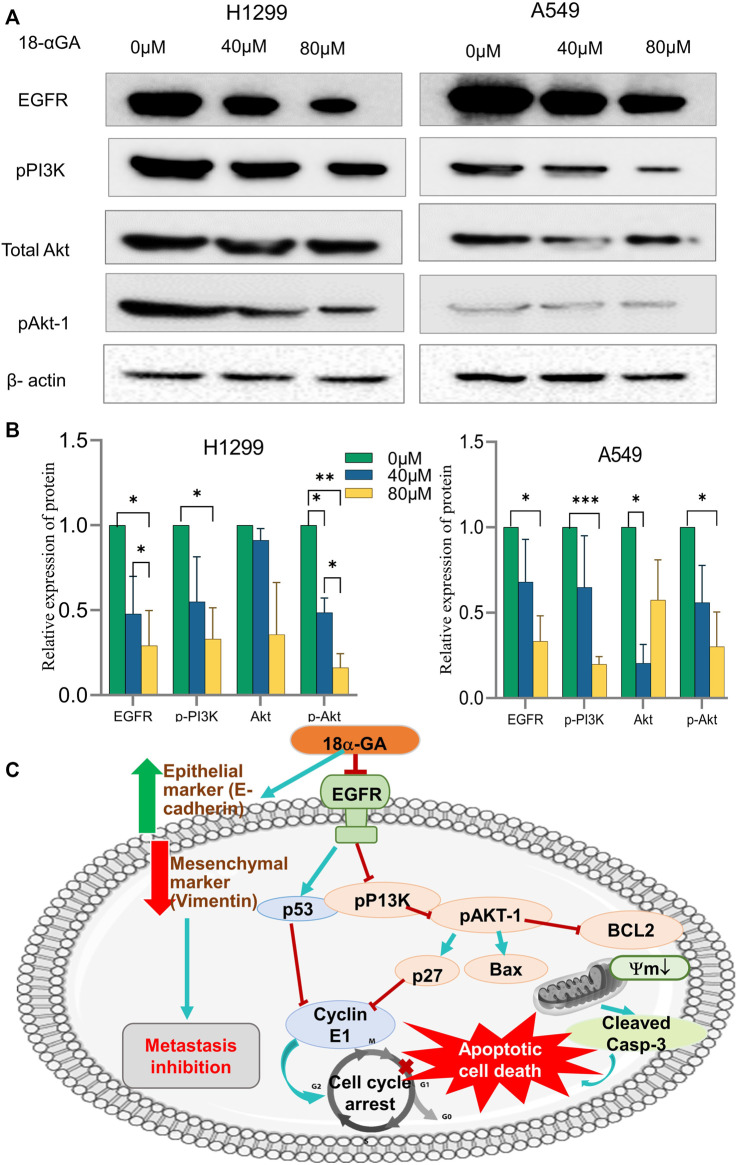
**(A)** Effect of different concentrations of 18α-GA treatment on the protein expression of EGFR, pPI3K, pan-Akt and pAKT1 in H1299 and A549 cells. **(B)** Graph representing relative expression of protein with respect to beta-actin in treated and untreated groups, statistical analysis was done by 2-way ANOVA followed by Tukey’s Post-hoc multiple comparison test, *p < 0.05, **p < 0.01, ***p < 0.001, ****p < 0.0001. **(C)** Depiction of the overall mechanism of action of 18α-GA against NSCLC as predicted by combining key targets found in the bioinformatics and *in-vitro* analysis.

The network pharmacology and pathway enrichment analysis predicted that EGFR, PI3K, and AKT1 was increasingly associated pathway with the anti-tumor properties of 18α-GA in NSCLC. Therefore, to confirm the *in-silico* findings, the protein expression levels of these targets were examined by western blotting. As shown in Figures 8A,B, the expression level of EGFR was apparently reduced in both the cell lines with increasing dose but the effect was more significant in H1299 cells as compared to A549 cells. Additionally, the phosphorylation levels of PI3K and Akt1 were significantly decreased after treatment with two concentrations of 18α-GA. These results validated that the highly expressed hub targets are repressed on treatment by 18α-GA and might regulate NSCLC proliferation and tumorigenesis *via* the EGFR-PI3K-Akt signaling pathway.

## 4 Discussion

The NSCLC, comprising adenocarcinoma, squamous cell carcinoma, and large-cell carcinoma subtypes, is the most aggressive and highly metastatic kind of lung cancer, leading to higher overall death rates than combined mortality rates of breast cancer, prostate cancer, colorectal cancer, and leukemia (Alexander et al., 2020). In an attempt to reduce lung cancer incidences and improve patients’ survival rates, great efforts are being made, including refinement of early-stage screening methods, understanding lung cancer etiology, and introducing newer state-of-the-art treatment regimens (Bade and dela Cruz, 2020). Recently neoadjuvant and adjuvant chemotherapy in conjunction with the current standard drug regimen (vandetanib, pazopanib, and axitinib) and radiotherapy have been instituted (Chen et al., 2020). However, these attempts are futile and somewhat ineffective in ameliorating the clinical outcome of NSCLC patients. To address this challenge, complementary therapeutic approaches have been the focus for the last few decades, including the use of natural compounds or medicinal plants to find novel therapeutic agents and new leads for synthetic or semi-synthetic chemotherapeutic drugs (Irshad and Husain, 2021; Yang et al., 2021). Not to forget, the lesser side-effects and toxicity associated with utilizing these agents in clini cal settings enhance patients’ quality of life and overall treatment outcomes. Countless such natural products have been under scrutiny for their chemopreventive properties, e.g., Curcumin and curcumin-derived semi-synthetic derivatives being administered to cancer patients that are proving as an adequate adjuvant remedy (Salehi et al., 2020; Ali et al., 2021). Nevertheless, there is always room to search for newer and better natural products from nature’s diverse sources that can be utilized to harness their chemopreventive capacities.

Although pentacyclic triterpenoids have a significant history of showing great potential as anti-cancer agents, there is still a gap in screening numerous triterpenoids present in nature (Markov et al., 2017; Salvador et al., 2017; Ghante and Jamkhande, 2019). Accordingly, in this study, the effect and mechanism of action of a pentacyclic triterpenoid compound- 18α-GA were evaluated on the growth, proliferation, metastasis, and survival of NSCLC by utilizing network pharmacology and *in-vitro* approaches. Systems pharmacology has become apparent as one of the most exciting approaches in investigating the mechanistic underpinnings ranging from molecular to tissue level of the natural products, medicinal plants, and traditional Chinese medicine against various diseases, including cancer (Kibble et al., 2015; Fang et al., 2017). Network pharmacology is a multi-disciplinary method and acts as the converging point of pharmacokinetics and pharmaco-mapping analysis, high-throughput omics data analysis, biological database retrieval, systems biology, drug repurposing, and structure-based drug designing (Chandran et al., 2017). It is a novel methodology that helps in creating and establishing “compound-target- pathway-disease” network models that aid in the prediction of disease targets, thereby increasing the efficiency of drug discovery and, in turn, snowballing the success rate of clinical trials with a simultaneous reduction in the expenditure associated with drug discovery (Li and Zhang, 2013).

Recently, targeted therapies have shown promise in chemoprevention and treatment, yet it has only been successfully utilized in a few cancer types (Charmsaz et al., 2018). The curative outcomes of single-target therapies are often counterweighed by either acquiring resistance or establishing compensatory signaling pathways in cancer cells. Network pharmacology breaks such limitations by deciphering the underlying putative multi-targeted, multi-channel therapeutic abilities of natural products and medicinal plants in treating heterogeneous diseases like cancer (Kibble et al., 2015). Thus, network pharmacology was utilized to systematically elucidate the mechanism of 18α-GA against NSCLC and further corroborate it by extensive *in-vitro* experiments. To the best of our knowledge, this study has first time delineated the anti-tumor effects of 18α-GA by reducing proliferation, inducing apoptosis, and inhibiting cell cycle progression as well as migration and metastasis in NSCLC cells.

At the outset of this study, the pharmacokinetics evaluation of the absorption, distribution, metabolism, excretion, and toxicity (ADME/T) confirmed the excellent druggability of 18α-GA *in vivo* (Supplementary Figure S5). The absorption parameter confirmed that 18α-GA is absorbed easily, and it follows Lipinski’s rule of drug-likeness hence, it is believed that 18α-GA has the characteristics to be developed into a drug. Subsequently, the MTT assay result verified that 18α-GA did not have a cytotoxic effect on HEK- 293 cells and had a negligible effect on its proliferative capacity. Meanwhile, network pharmacological analysis established at least 181 potential targets of 18α-GA against NSCLC. Further evaluation of PPI network that had 181 nodes and 964 edges was done by recognising the Top 20 targets on the basis of Degree, Betweenness, Closeness and MCC values. According to these network topological properties, EGFR, PI3KR1, AKT1, MAPK1, SRC, and IGF1 were identified as the core targets of anti-NSCLC effects of 18α-GA; most of these targets have critical roles in cell proliferation, survival, cell cycle regulation, metastasis, and invasion.

These targets were chosen as they were the key nodes of the PPI network and the binding energies confirmed that among these 6 key targets, EGFR and AKT1 had the most excellent affinity toward our compound. In the docking studies, lower scores correspond to lower matching energies, which means that there is a higher likelihood of contact and a more stable conformation of ligand-receptor binding. The results showed that the binding energy of these two proteins specifically were less than −7.5 kcal/mol indicating strong binding activity.

Consistent with this result, the GO analysis of common targets of 18α-GA and NSCLC displayed several important biological processes, molecular functions and cellular compartments. The highly significant biological processes when screened included ErbB (EGFR) signaling pathway, cell proliferation, regulation of PI3K signalling, protein tyrosine kinase signalling, regulation of apoptosis and phosphatidyl-inositol signalling. Similarly, the nucleus, plasma membrane, mitochondrial matrix, cytoplasm are the few important cellular components that are involved. The molecular functions gene ontology disclosed the contribution protein kinase binding, protein kinase activity, protein phosphatase binding, insulin receptor binding, phosphatidylinositol-4,5-bisphosphate 3-kinase activity as the crucial effecters of 18α-GA targets. The results of KEGG analysis revealed that the highly enriched top pathways involved were the PI3K-Akt signaling pathway, ErbB (EGFR) signaling pathway, pathways in cancer, estrogen signaling pathway, and Ras signaling pathway, among other closely related cancer signaling pathways associated with targets of 18α-GA against NSCLC. The GO terms and KEGG pathways allow for concrete prediction of the mechanisms involved in anti-cancer activity of the compound by taking into considerations the large-scale molecular datasets produced by genome sequencing and other high-throughput experimental techniques. Taken together result of the above *in-silico* analysis, it can be inferred that 18α-GA may exert its therapeutic effects on NSCLC by regulating ErbB signaling and PI3K/AKT signaling pathway, which is widely involved in tumor proliferation, apoptosis, and metastasis regulation. EGFR belongs to the protein kinase superfamily, a member of the ErbB family, and is a transmembrane glycoprotein and a receptor of epidermal growth factor. Mostly NSCLC harbors EGFR mutations and is widely involved in causing tumorigenesis (da Cunha Santos et al., 2011). EGFR is a vital driver mutation because it regulates a multitude of intracellular signaling networks. Overexpression of EGFR can lead to unregulated activation of its downstream signaling pathways, which include PI3K/AKT, Ras-ERK/MAP kinase, Src, JAK/STAT, and PKC, thereby leading to uncontrolled proliferation, survival, migration of invasion of cancer cells (Seshacharyulu et al., 2012). Similarly, PI3K/AKT is correlated with the initiation and development of cancer which is conducive to the proliferation, cell cycle progression, migration, and invasion of cancer cells. This pathway is most commonly and consistently activated in human cancers and is closely associated with tumorigenesis, proliferation, apoptosis, metastasis, epithelial-mesenchymal transition (EMT), metabolism, and chemoresistance. Hence, it is no surprise that these pathways have been attractive targets for anti-cancer therapies (Chang et al., 2003). Thus, this indicates that inhibition of these signaling molecules might help in suppressing cancer cell growth and tumorigenesis.

The data obtained as stated above was validated in cell culture to confirm the effect of 18α-GA on NSCLC cancer cell lines; H1299 and A549. It was observed that 18α-GA could inhibit cell growth and was found to be cytotoxic for both the cell lines at 24 and 48 h of treatment. Although, the growth inhibitory effect was more pronounced at 48 h. Thus, two fixed dosages were adopted for the treatment of 48 h in this study. As uncontrolled cell cycle progression supports the growth of cancer cells, the effect of 18α-GA was found that it was able to arrest the cell cycle at the G1 phase, hence inhibiting the G1-S transition. This was in accordance with the expressions of key regulatory genes p27, Cyclin E1, and p53 of this transition (Raghunandhakumar et al., 2013). It is important to note here that these genes are also a part of the signaling network identified by bioinformatics analysis in this study. EGFR stimulates the PI3K/AKT axis which directly affects the expression of p27 and p21 which in turn regulated cyclin E1 expression and act in the G1/S transition (Chang et al., 2003). The activated Akt negatively regulates p27 and p21 decreasing their expression and promoting the cell cycle. Accordingly, it was observed that the expression of p27 was increased with a concomitant decrease in Cyclin E1.

The FACS and morphological analysis (AO/EB) in this study suggested that the anti-tumor effect of 18α-GA against NSCLC cell lines, H1299 and A549 was found to be associated with the induction of apoptotic cell death. Additionally, the protein expression analysis by western blotting revealed that apoptosis was induced after treatment with 18α-GA due to a decrease in the expression of Bcl-2 with a concomitant increase in the expressions of Bax and cleaved caspase- 3 proteins. The Bcl2 family of proteins maintains a balance between pro and anti-apoptotic proteins and is downstream of PI3K/AKT signaling pathway, hence, the unchecked pathway disturbs this balance and causes the uncontrolled proliferation (Pilling and Hwang, 2019). Bcl-2 and Bax are two distinct members of Bcl-2 family that regulates cellular apoptosis and an elevated ratio of Bax/Bcl-2 is known to induce apoptosis (Perlman et al., 1999) which was seen in our data too. There are other reports suggesting that the increased ratio of Bax/Bcl-2 could also increase caspase- 3 activation and irreversibly activate the process of apoptosis in cells (Salakou et al., 2007). It has been reported that the overactive EGFR- PI3K/AKT pathway plays a vital role in perturbing the apoptotic system of cancer cells and thus evading this process altogether and targeting these pathway proteins can induce apoptosis (Nicholson and Anderson, 2002; Jeon et al., 2016).

Metastatic dissemination is an important hallmark of cancer transformation which involves Epithelial to Mesenchymal transition (EMT) causing the cells to lose epithelial markers and gain mesenchymal properties (Huber et al., 2005; Bhat et al., 2014; Duan et al., 2014). Congruently, this study showed increased expression of E-cadherin, an epithelial marker with a simultaneous decrease of Vimentin expression which is a mesenchymal marker after treatment with 18α-GA. Moreover, a significant reduction in the wound healing process was evident in NSCLC cell lines after 18α-GA incubation, therefore it may be speculated that 18α-GA could inhibit EMT and hence metastasis. In addition, the protein expression levels of key signaling molecules i.e., EGFR, pPI3K, and pAkt-1 were validated through western blotting. It was observed that 18α-GA treated NSCLC cells showed significantly reduced expression of these targets indicating that NSCLC inhibition was mainly *via* the regulation of cell proliferation and survival through the EGFR- PI3K/Akt signaling pathways.

In summary, the therapeutic efficacy of 18α-GA against NSCLC was explored by bio-informatics and confirmed using *in-vitro* studies. The present study confirms the anti-tumorigenic effects of 18α-GA on NSCLC, thereby inhibiting proliferation and metastasis of lung cancer cells by inducing apoptosis and inhibiting cell cycle progression, EMT, and regulating EGFR-PI3K/AKT signaling pathway. It was also reported that other closely related pathways may be modulated by 18α-GA to suppress NSCLC growth as assessed by network pharmacological and functional enrichment studies that need future *in-vitro* investigations. The predicted model of the mechanism of action of 18α-GA in restraining NSCLC growth and progression has been elucidated in Figure 8C.

## Conclusion

In this study, an integrative approach involving network pharmacology and experimental assays was utilized to explore the underlying anti-NSCLC mechanisms of a natural compound, 18α-GA for the first time. The antiproliferative effect of 18α-GA on two NSCLC cell lines was due to inhibition of cell growth by induction of apoptosis, arrest of cell cycle progression, reduction in the migratory potential, and regulation of EGFR- PI3K/Akt pathway. Moreover, network pharmacology analysis suggested the involvement of multiple pathways and molecular targets of 18α-GA, hence forming a base of its multi-targeted efficacy against NSCLC. Although a comprehensive approach was utilized in this study, further investigation may provide more insight using animal models.

## Data Availability

The original contributions presented in the study are included in the article/Supplementary Material. Further inquiries can be directed to the corresponding author.
