# Supplementary material for: Integrated network pharmacology and experimental analysis unveil multi-targeted effect of 18α- glycyrrhetinic acid against non-small cell lung cancer
Source: Front Pharmacol. 2022 Oct 12;13:1018974. doi: 10.3389/fphar.2022.1018974 (PMC9596789; doi:10.3389/fphar.2022.1018974)

Supplementary figure 1: Overall framework of the study.

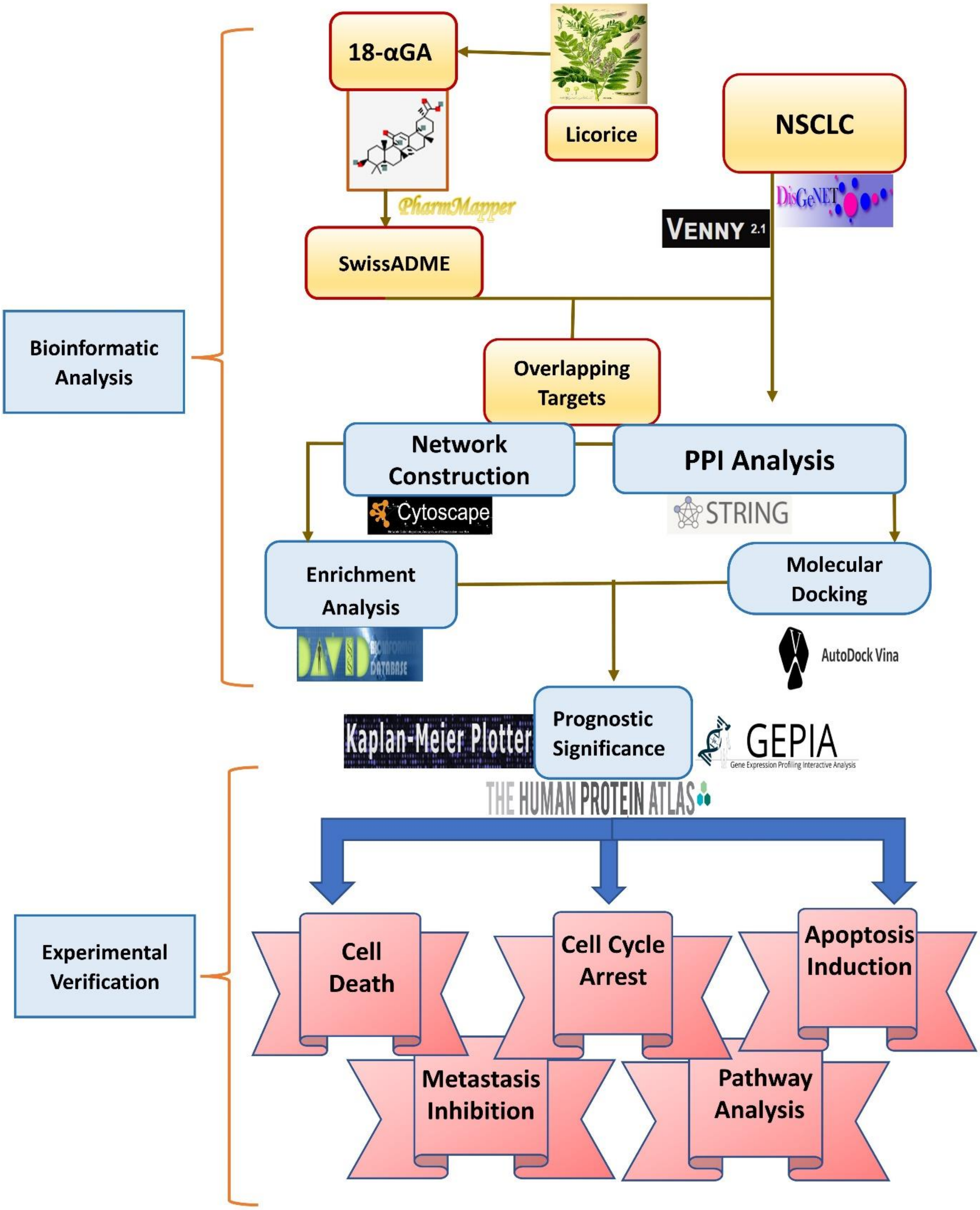

Supplementary figure 2: 18-aGA STRING PPI network.

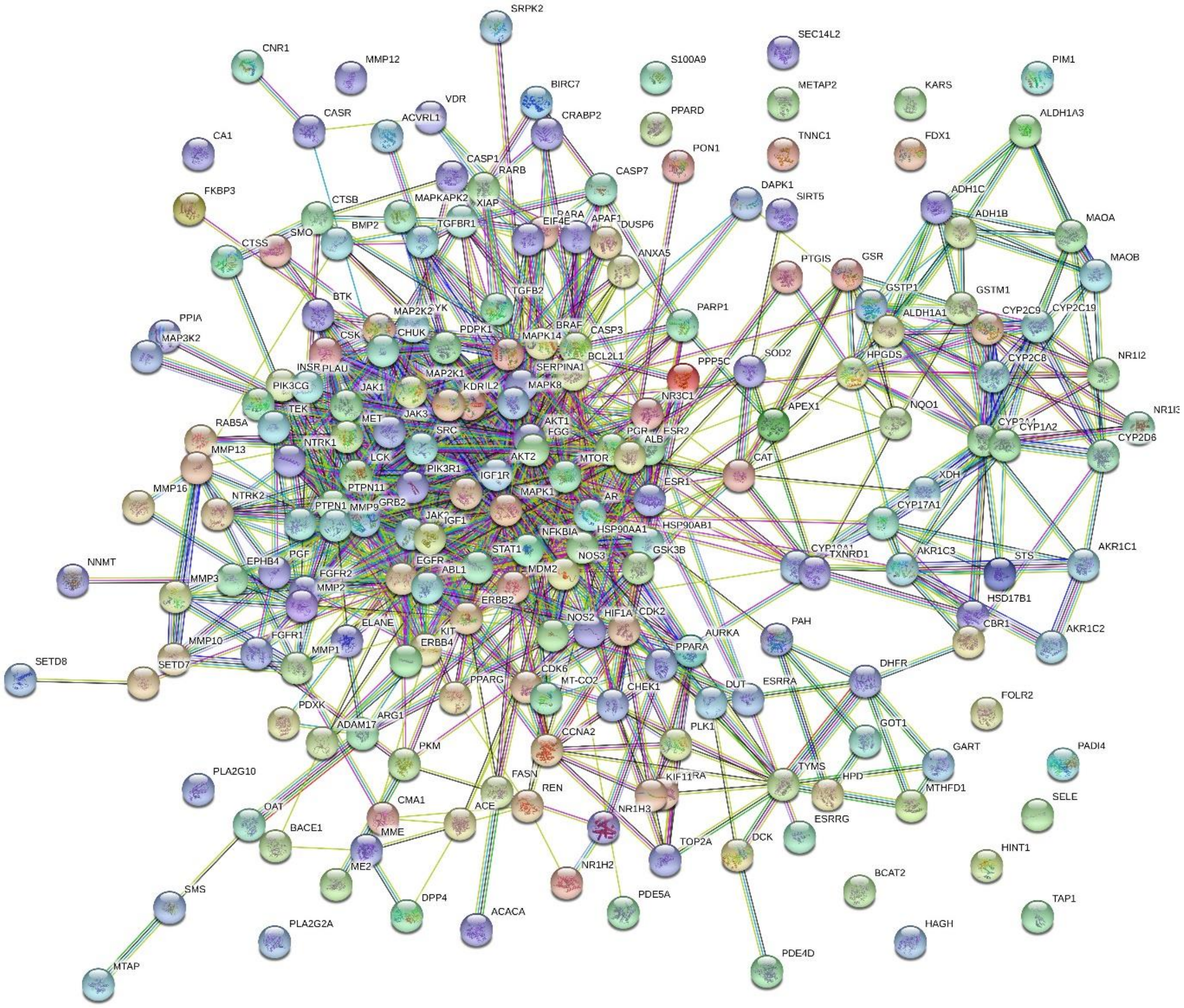

Supplementary figure 3: External validation of mRNA and protein expression and prognosis value of core targets 0f 18-αGA against NSCLC. **(A)** Boxplot of mRNA expression of core genes using GEPIA. These red box plots represent LUSD and LUAD samples; grey boxplots represent normal lung samples. **(B)** The representative protein expression of the core proteins in NSCLC tissue and normal tissue from the immunohistochemistry data from the HPA database. **(C)** The prognostic value of the expression of the 6 hub genes. The survival data were analyzed by the Kaplan-Meier Plotter database P < 0:05). Patients with expression above the median are indicated in the red line, while the black line represents expression below the median. HR represents the hazard ratio.

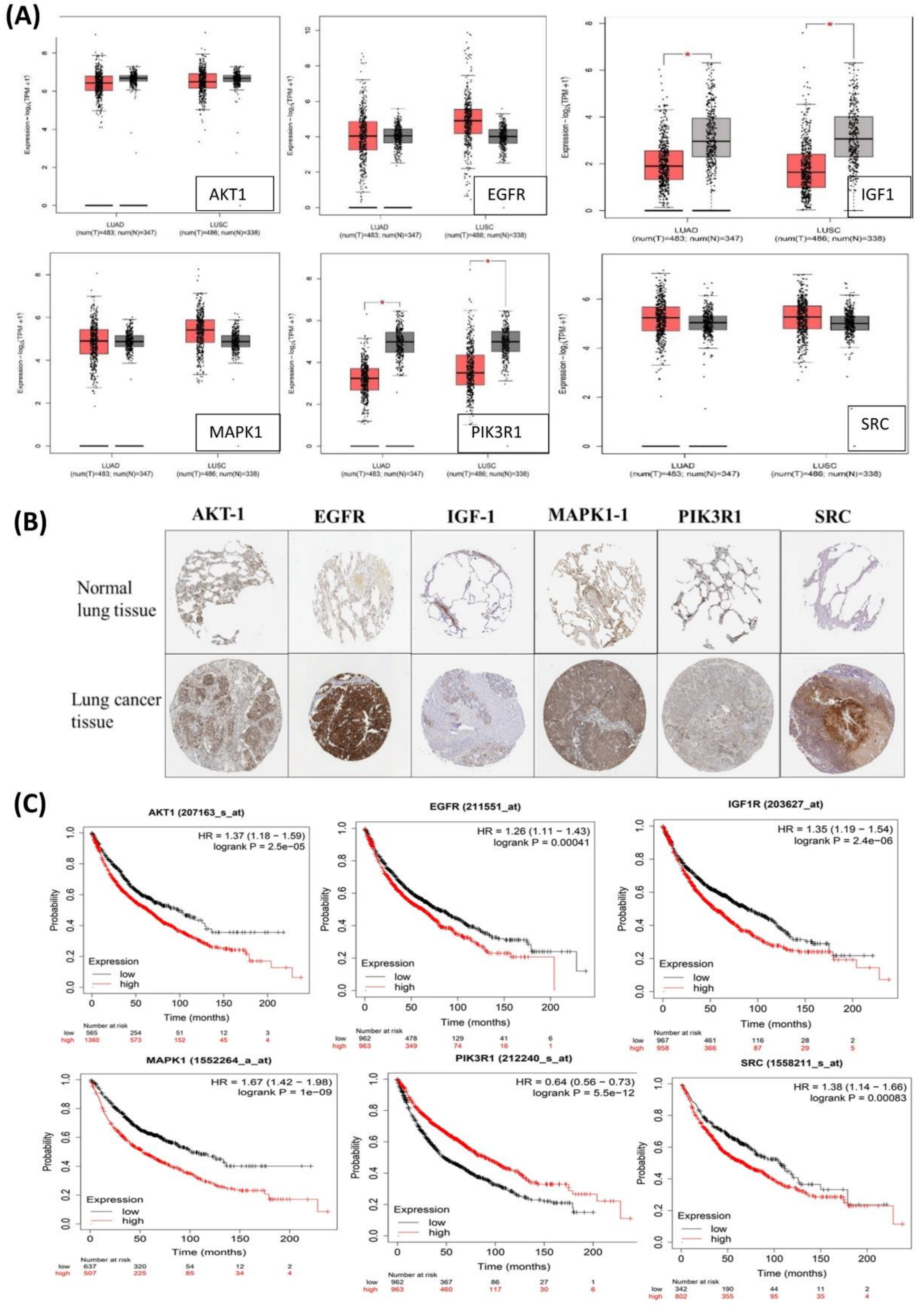
(A)
AKT1
EGFR
IGF1
MAPK1
PIK3R1
SRC
Expression - log2(TPM +1)
LUAD
(num(T)=483; num(N)=347)
LUSC
(num(T)=486; num(N)=338)
(B)
AKT-1
EGFR
IGF-1
MAPK1-1
PIK3R1
SRC
Normal lung tissue
Lung cancer tissue
(C)
AKT1 (207163_s_at)
HR = 1.37 (1.18 − 1.59)
logrank P = 2.5e−05
EGFR (211551_at)
HR = 1.26 (1.11 − 1.43)
logrank P = 0.00041
IGF1R (203627_at)
HR = 1.35 (1.19 − 1.54)
logrank P = 2.4e−06
MAPK1 (1552264_a_at)
HR = 1.67 (1.42 − 1.98)
logrank P = 1e−09
PIK3R1 (212240_s_at)
HR = 0.64 (0.56 − 0.73)
logrank P = 5.5e−12
SRC (1558211_s_at)
HR = 1.38 (1.14 − 1.66)
logrank P = 0.00083
Probability
Time (months)
Expression
low
high
Number at risk

Supplementary figure 4: Graph representing %cytotoxicity of 18αGA against HEK-293 cell line.

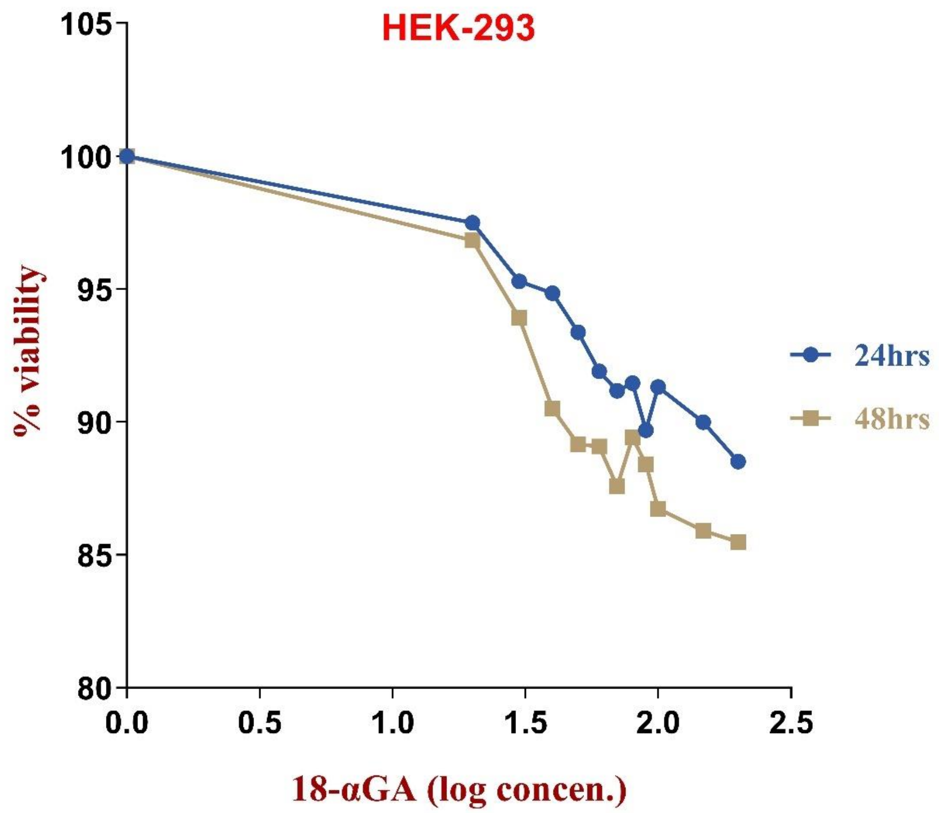

Supplementary figure 5: image representing ADMET properties of 18-αGA evaluated using SWISS-ADME.

Molecule 1

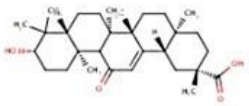

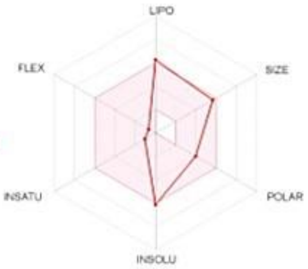

SMILES O=C1C=C2[C@H]3C[C@](C)(CC[C@]3(C)CC[C@]2([C@]2(C1[C@@]1(C)CC[C@@H](C([C@@H]1CC2)(C)C)O)C)C)C(=O)O

Physicochemical Properties	
Formula	C30H46O4
Molecular weight	470.68 g/mol
Num. heavy atoms	34
Num. arom. heavy atoms	0
Fraction Csp3	0.87
Num. rotatable bonds	1
Num. H-bond acceptors	4
Num. H-bond donors	2
Molar Refractivity	136.85
TPSA	74.60 Å²

Lipophilicity	
Log $P_{o/w}$ (iLOGP)	3.45
Log $P_{o/w}$ (XLOGP3)	5.49
Log $P_{o/w}$ (WLOGP)	6.41
Log $P_{o/w}$ (MLOGP)	4.87
Log $P_{o/w}$ (SILICOS-IT)	5.55
Consensus Log $P_{o/w}$	5.15

Water Solubility	
Log *S* (ESOL)	-6.15
Solubility	3.32e-04 mg/ml ; 7.06e-07 mol/l
Class	Poorly soluble
Log *S* (Ali)	-6.81
Solubility	7.21e-05 mg/ml ; 1.53e-07 mol/l
Class	Poorly soluble
Log *S* (SILICOS-IT)	-6.00
Solubility	4.75e-04 mg/ml ; 1.01e-06 mol/l
Class	Moderately soluble

Pharmacokinetics	
GI absorption	High
BBB permeant	No
P-gp substrate	Yes
CYP1A2 inhibitor	No
CYP2C19 inhibitor	No
CYP2C9 inhibitor	No
CYP2D6 inhibitor	No
CYP3A4 inhibitor	No
Log K_p (skin permeation)	-5.27 cm/s

Druglikeness	
Lipinski	Yes; 1 violation: MLOGP>4.15
Ghose	No; 3 violations: WLOGP>5.6, MR>130, #atoms>70
Veber	Yes
Egan	No; 1 violation: WLOGP>5.88
Muegge	No; 1 violation: XLOGP3>5
Bioavailability Score	0.85

Medicinal Chemistry	
PAINS	0 alert
Brenk	0 alert
Leadlikeness	No; 2 violations: MW>350, XLOGP3>3.5
Synthetic accessibility	6.08

Supplementary figure 6: Gel images of figure 5C. images in panel 3 used in the main article.

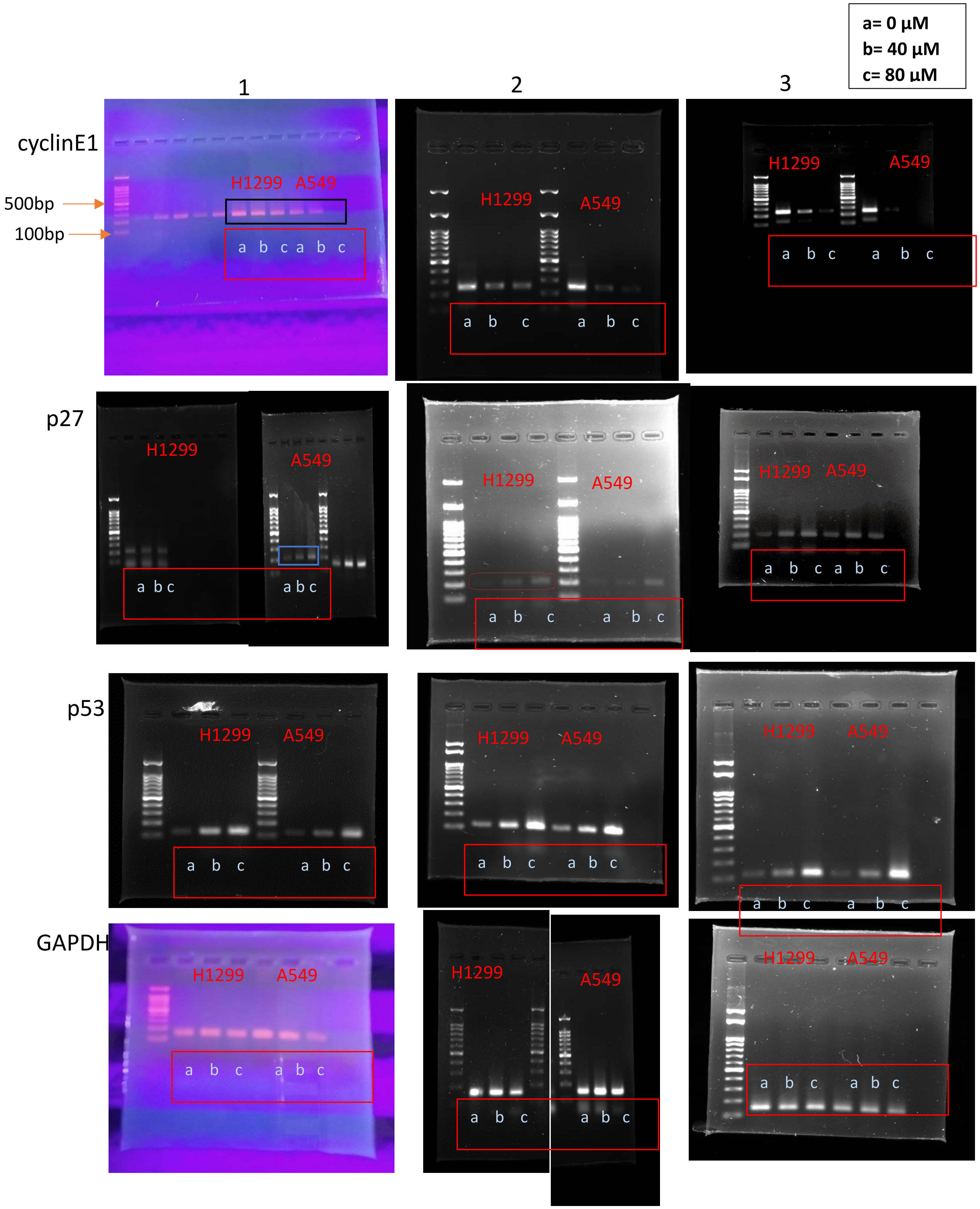

Supplementary figure 7: Gel images of figure 7C. images in panel 3 used in the main article

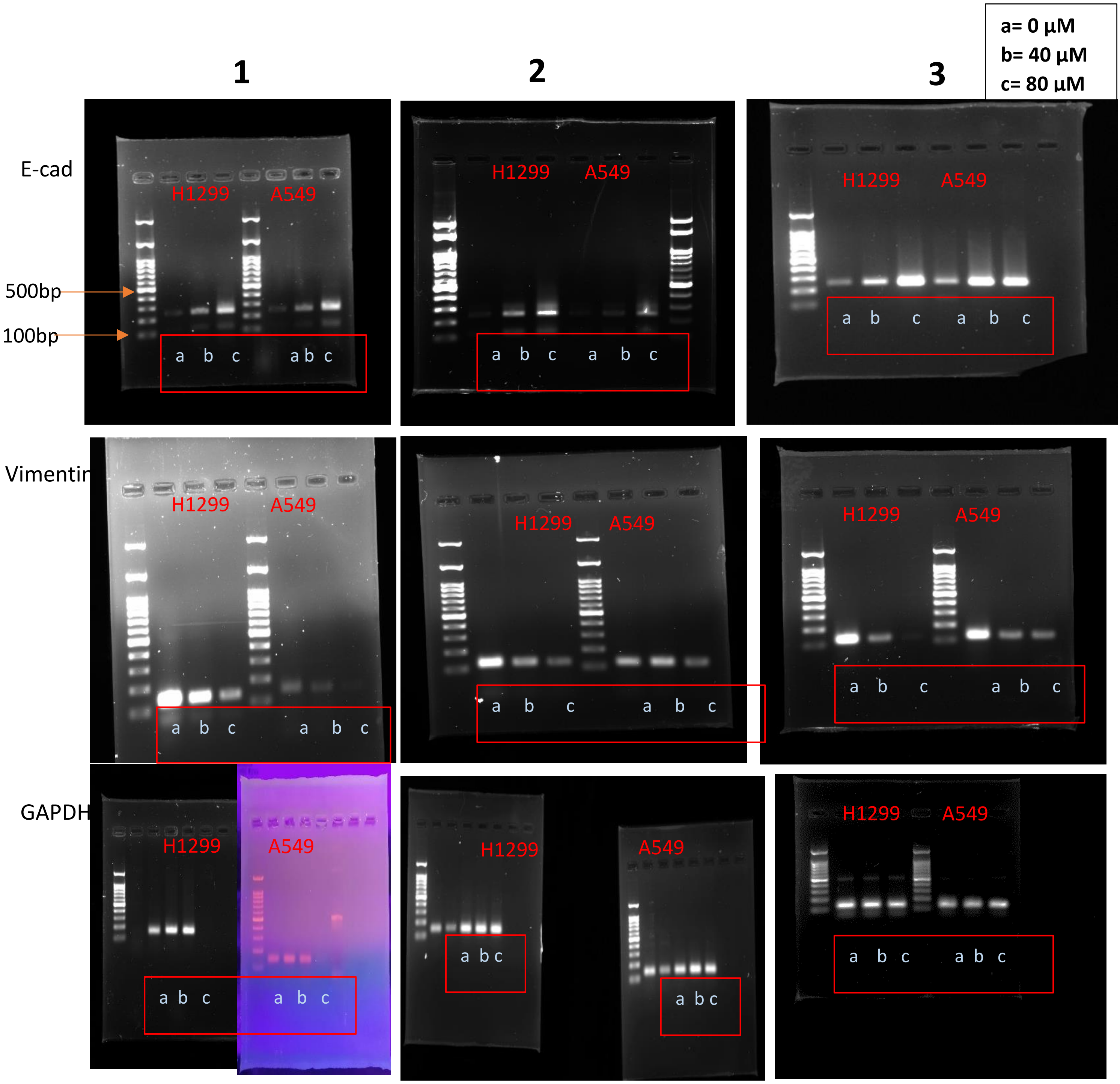

Supplementary figure 8: Blot/ x-ray images of figure 6C. Images in panel 3 used in the main figure

1

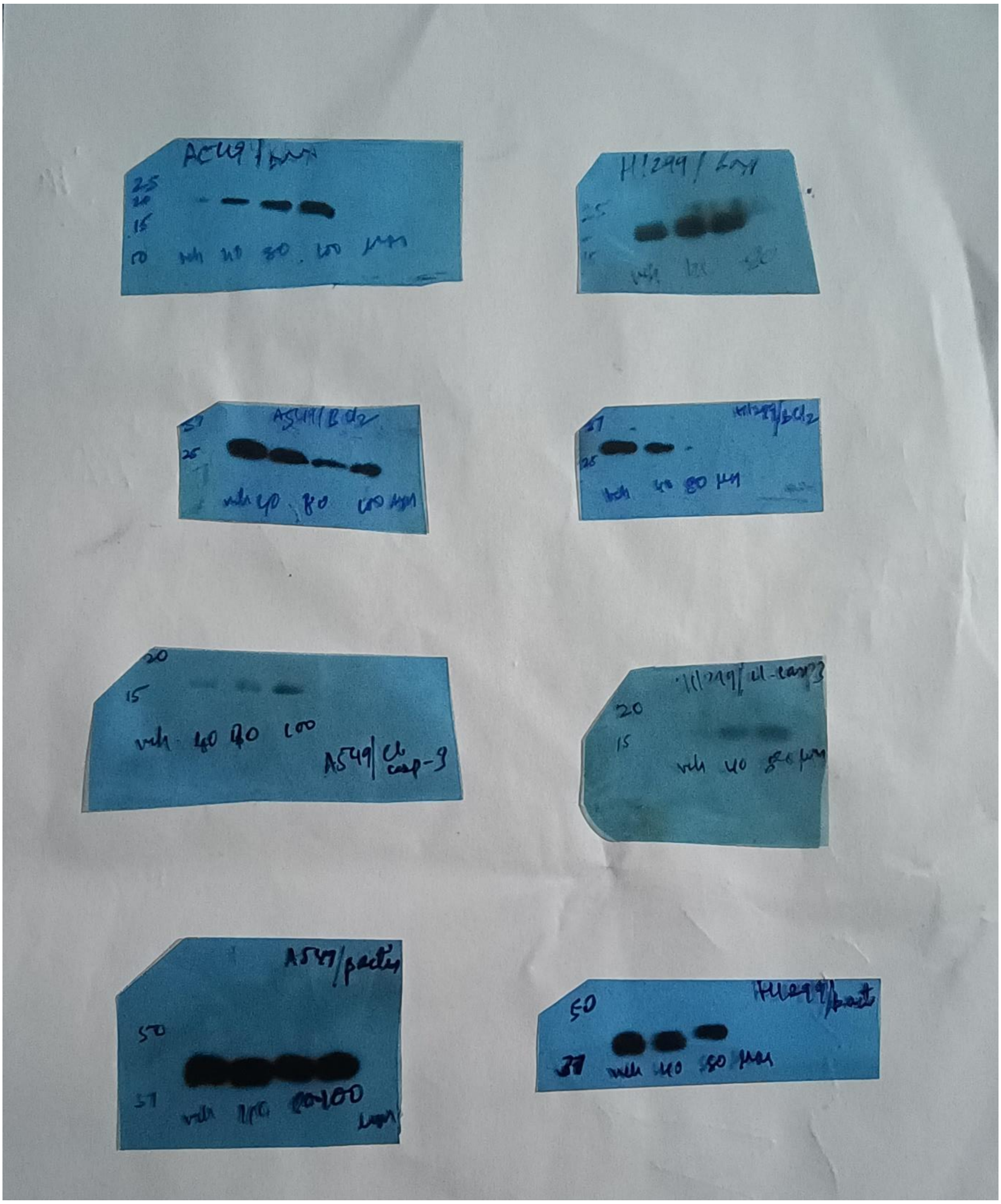

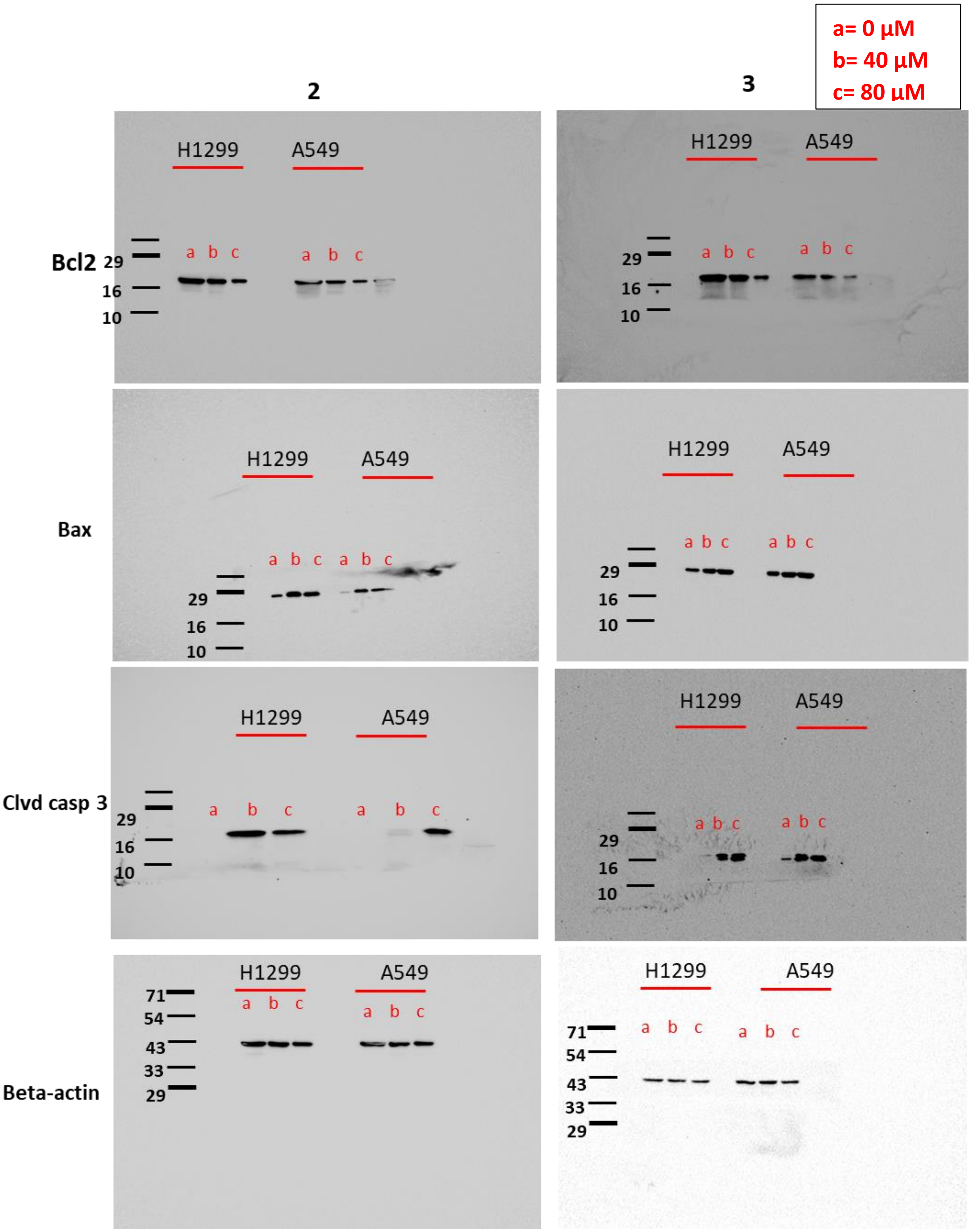
a= 0 µM
b= 40 µM
c= 80 µM
2
3
Bcl2
Bax
Clvd casp 3
Beta-actin
H1299
A549
a b c
29
16
10
71
54
43
33

Supplementary figure 9: Blot/ xray images of figure 8A. Images in panel 3 used in main figure

1

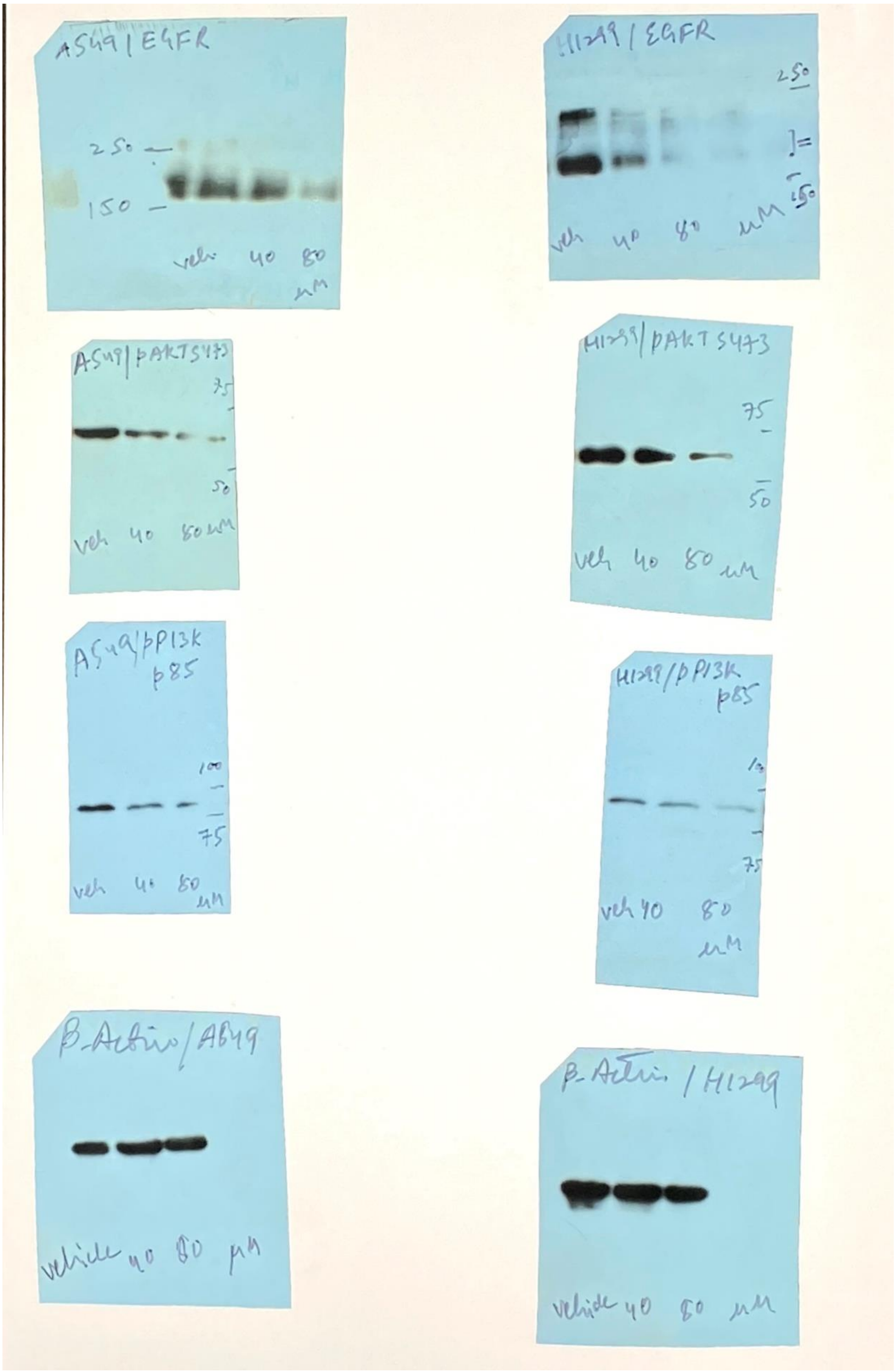

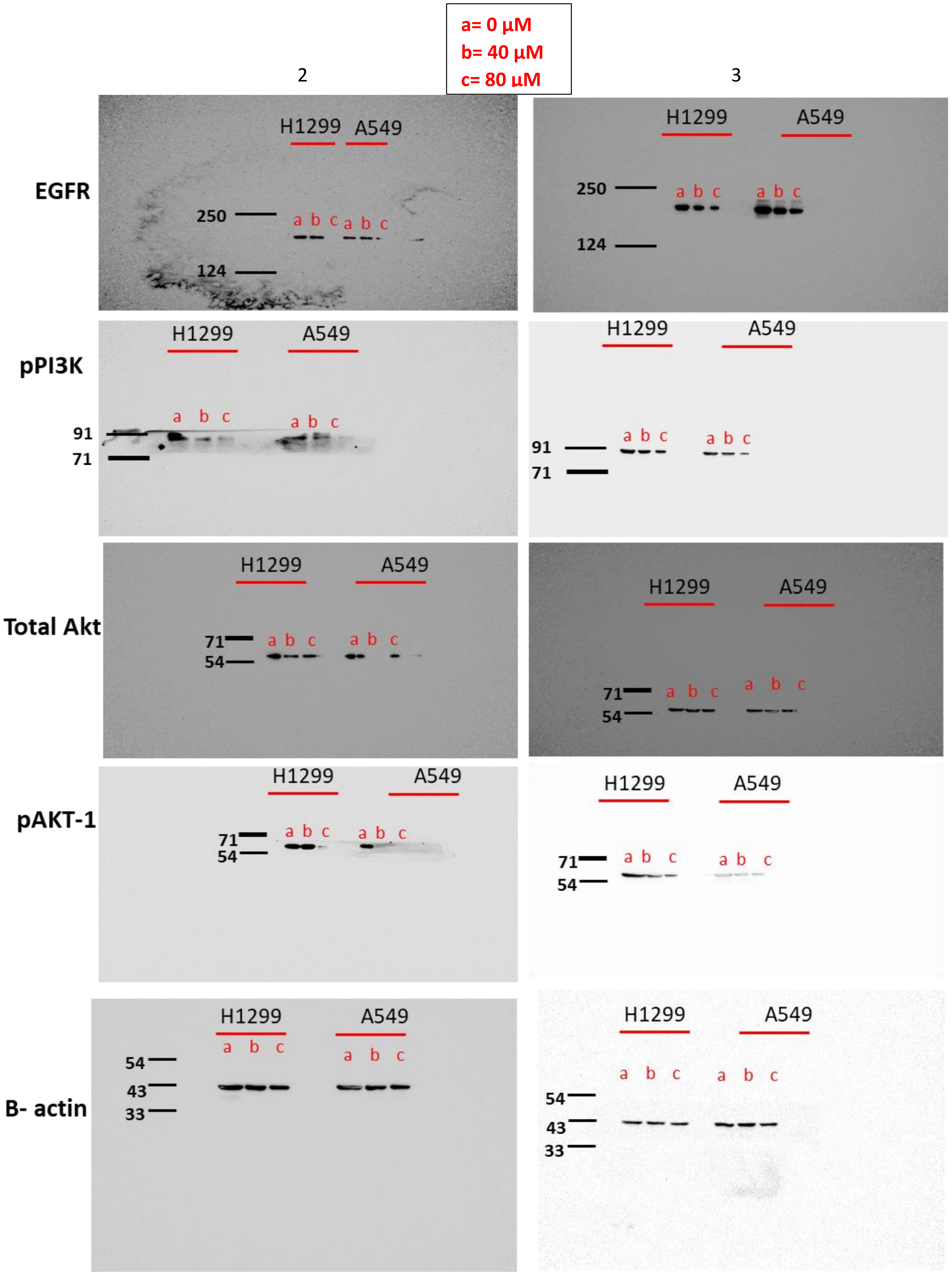
a= 0 µM
b= 40 µM
c= 80 µM
2
3
EGFR
pPI3K
Total Akt
pAKT-1
B- actin
H1299
A549
a b c
250
124
91
71
54
43
33

Supplementary Figure 10: original blot image of total akt

1

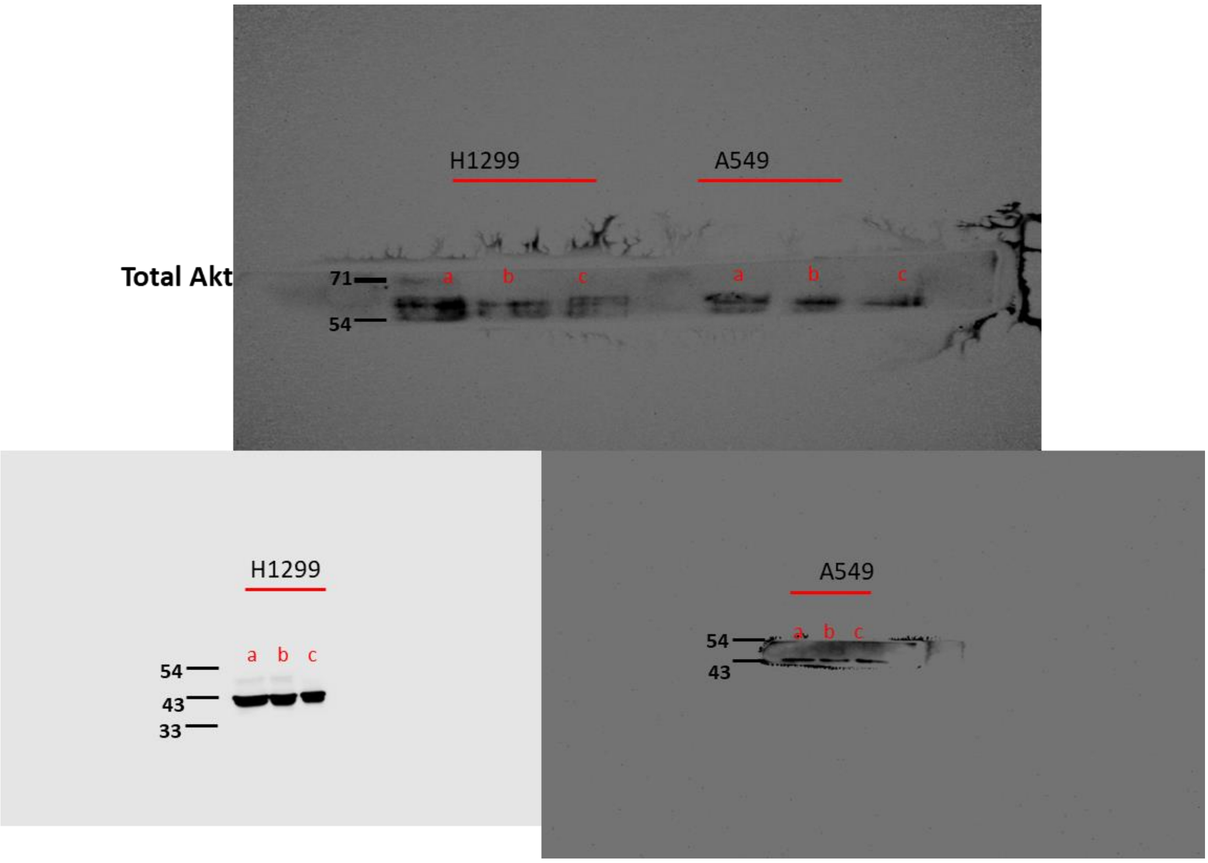

Supplement: Supplementary file 1 [file DataSheet1.PDF]
