# Supplementary material for: Integrated network pharmacology and experimental analysis unveil multi-targeted effect of 18α- glycyrrhetinic acid against non-small cell lung cancer
Source: Front Pharmacol. 2022 Oct 12;13:1018974. doi: 10.3389/fphar.2022.1018974 (PMC9596789; doi:10.3389/fphar.2022.1018974)
Supplement: Supplementary file 2 [file DataSheet2.ZIP › Supplementary Table 1. Details of primers used in the study.docx]

Supplementary Table 1. Details of primers used in the study.

| Primer Name | Forward (5’-3’) | Reverse (5’-3’) | Product Size |
| --- | --- | --- | --- |
| Cyclin E1 | GTGCAAGCCTCGGATTATT | GTGCTGATCCCTTAAGTATGTC | 247 |
| p27 | AAGGGCCAACAGAACAGAAG | GGATGTCCATTCAATGGAGTC | 213 |
| P53 | CCTCAGCATCTTATCCGAGTGG | TGGATGGTGGTACAGTCAGAGC | 128 |
| E-cadherin | CGACAAAGGACAGCCTATTT | CGACAAAGGACAGCCTATTT | 250 |
| Vimentin | CCTGCAATCTTTCAGACAGG | CTCCTGGATTTCCTCTTCGT | 127 |
| GAPDH | GACAGTCAGCCGCATCTTC | CAACAATATCCACTTTACCAG | 150 |
