# Supplementary material for: Integrated network pharmacology and experimental analysis unveil multi-targeted effect of 18α- glycyrrhetinic acid against non-small cell lung cancer
Source: Front Pharmacol. 2022 Oct 12;13:1018974. doi: 10.3389/fphar.2022.1018974 (PMC9596789; doi:10.3389/fphar.2022.1018974)
Supplement: Supplementary file 2 [file DataSheet2.ZIP › Supplementary Table 2. Docking scores of 18a-GA with different target proteins.docx]

**Supplementary Table 2.** Docking scores of 18α-GA with different target proteins.

| **Target protein (PDB)** | **Grid dimension of pocket** | **Docking score (*kcal/mol*.)** | ***Pki* (binding constant)** | **Ligand efficiency** | **Hydrogen bonding interactions** | **Distance**  **(Å)** | **Hydrophobic interactions** |
| --- | --- | --- | --- | --- | --- | --- | --- |
| **1DNQ**  (EGFR) | Grid Box Center  Coordinates  center x = -5 .506  center_y = - 10.091  center_z = -8.317  Grid Box Size  size_x = 88  size_y = 74  size_z = 68 | -8.8 | -6.45 | 0.226 | Arg231 NH2…. O35  THR266 OG1…. O1  ASP232 OD1…. H34  LYS260 NH…. O33  LYS260 NH…. O32 | 2.84  2.97  2.10  3.06  3.18 | **Alkyl**  Val6, Lys5, Arg231, Val268 |
| **1PME**  (MAPK1) | center_x = -4.428  center_y = 8.76  center_z = 47.403  size_x = 76  size_y = 58  size_z = 81 | -8.2 | 6.01 | 0.21 | Asp111 OD2.... H36  Asp167 OD1.... H34  Gly169 CA.... O33 | 2.21  2.12  3.54 | **Alkyl**  Tyr36  **Pi-alkyl**  Lys54, Val39 |
| **1H10**  (AKT1) | center_x = 21.606  center_y = 14.477  center_z = 10.021  size_x = 55  size_y = 52  size_z = 64 | -7.9 | 5.79 | 0.203 | Ser56 OG…. H34  Ser56 OG…. O1  Trp80 N…. O32 | 2.03  3.01  3.08 | **Alkyl**  Leu110, Ala58 |
| **1A07**  (SRC) | center_x = 44.788  center_y = 18.064  center_z = 19.231  size_x = 53  size_y = 44  size_z = 53 | -7.1 | 5.21 | 0.182 | Arg208 H…. O32 | 2.32 | **Alkyl**  Tyr205, Tyr216, Arg208 |
| **1BFI**  **(**PI3KR1**)** | center_x = 87.374  center_y = 0.972  center_z = 0.552  size_x = 62  size_y = 102  size_z = 74 | -6.8 | 4.99 | 0.174 | His84 NE2….H34 | 2.13 | **Pi-alkyl**  His84 |
| **1B9G**  (IGF1) | center_x = 87.374  center_y = 0.972  center_z = 0.552  size_x = 62  size_y = 102  size_z = 74 | -6.7 | 4.91 | 0.172 | NA | NA | **Pi-sigma**  Tyr24  **Pi-alkyl**  Tyr24 |
