# Supplementary figures and images for: Integrated network pharmacology and experimental analysis unveil multi-targeted effect of 18α- glycyrrhetinic acid against non-small cell lung cancer
Source: Front Pharmacol. 2022 Oct 12;13:1018974. doi: 10.3389/fphar.2022.1018974 (PMC9596789; doi:10.3389/fphar.2022.1018974)

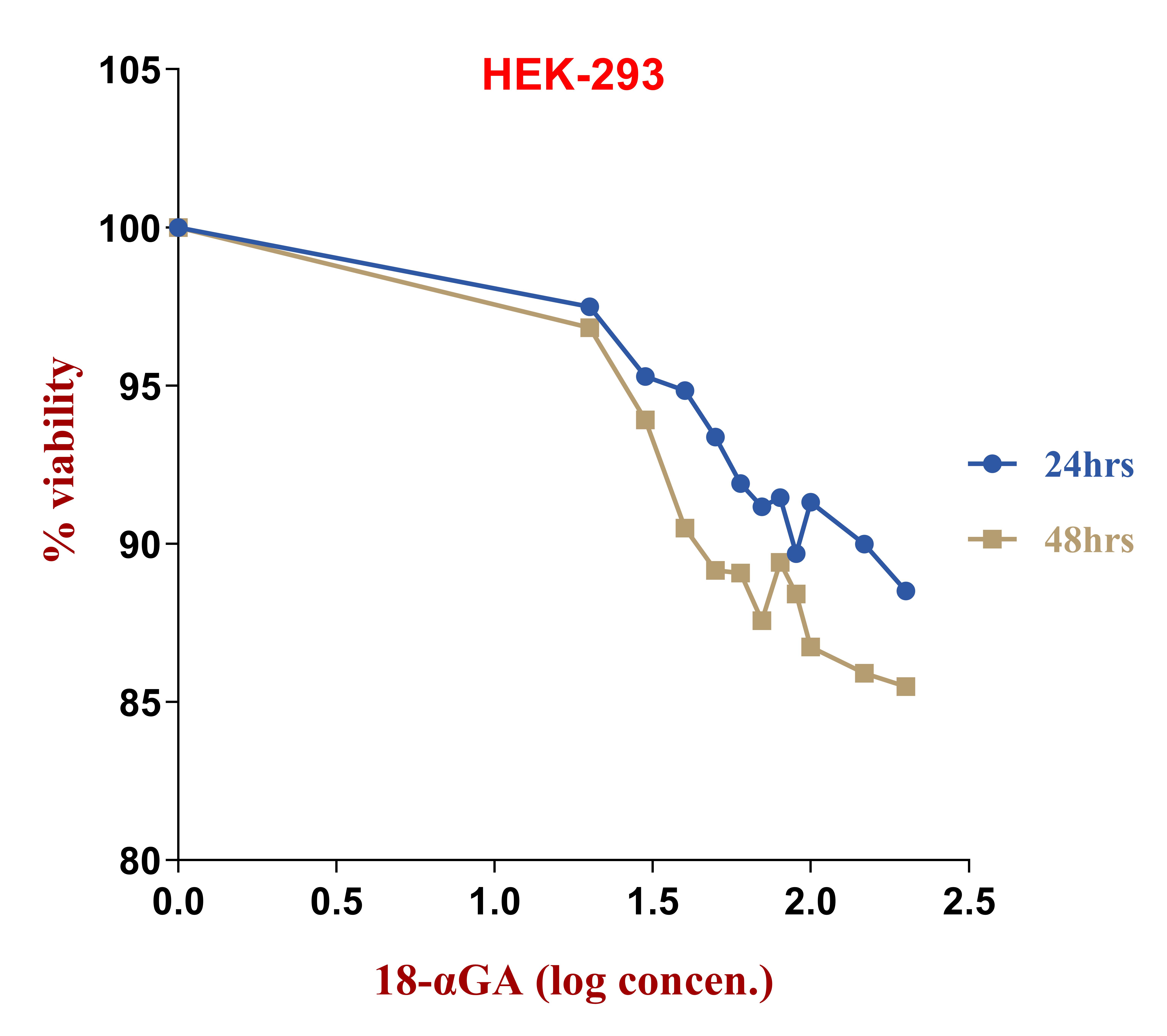

Supplement: Supplementary file 2 [file DataSheet2.ZIP › Supplementary Fig. 4 (HEK 293 MTT assay).jpg]

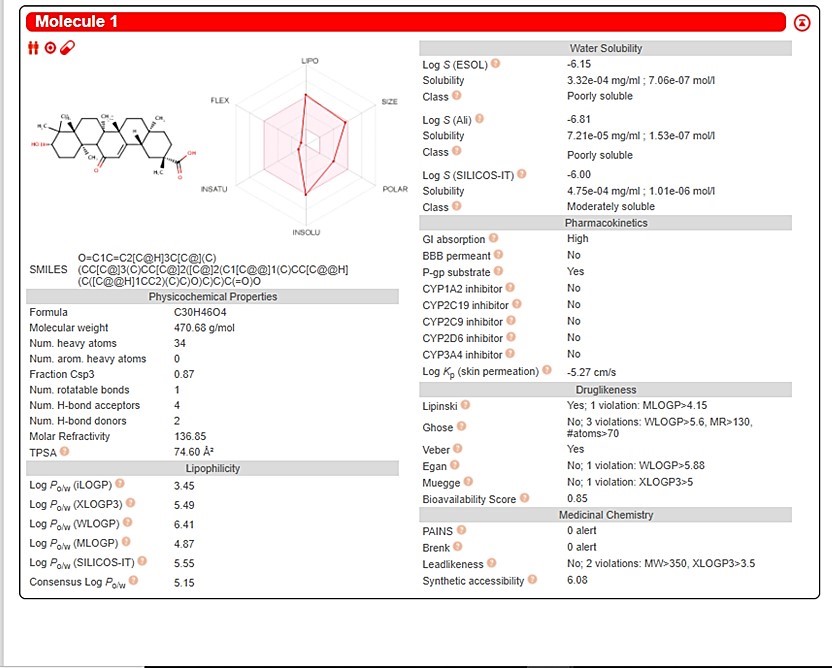

Supplement: Supplementary file 2 [file DataSheet2.ZIP › Supplementary Fig. 5 (ADMET properties of 18-aGA).jpg]

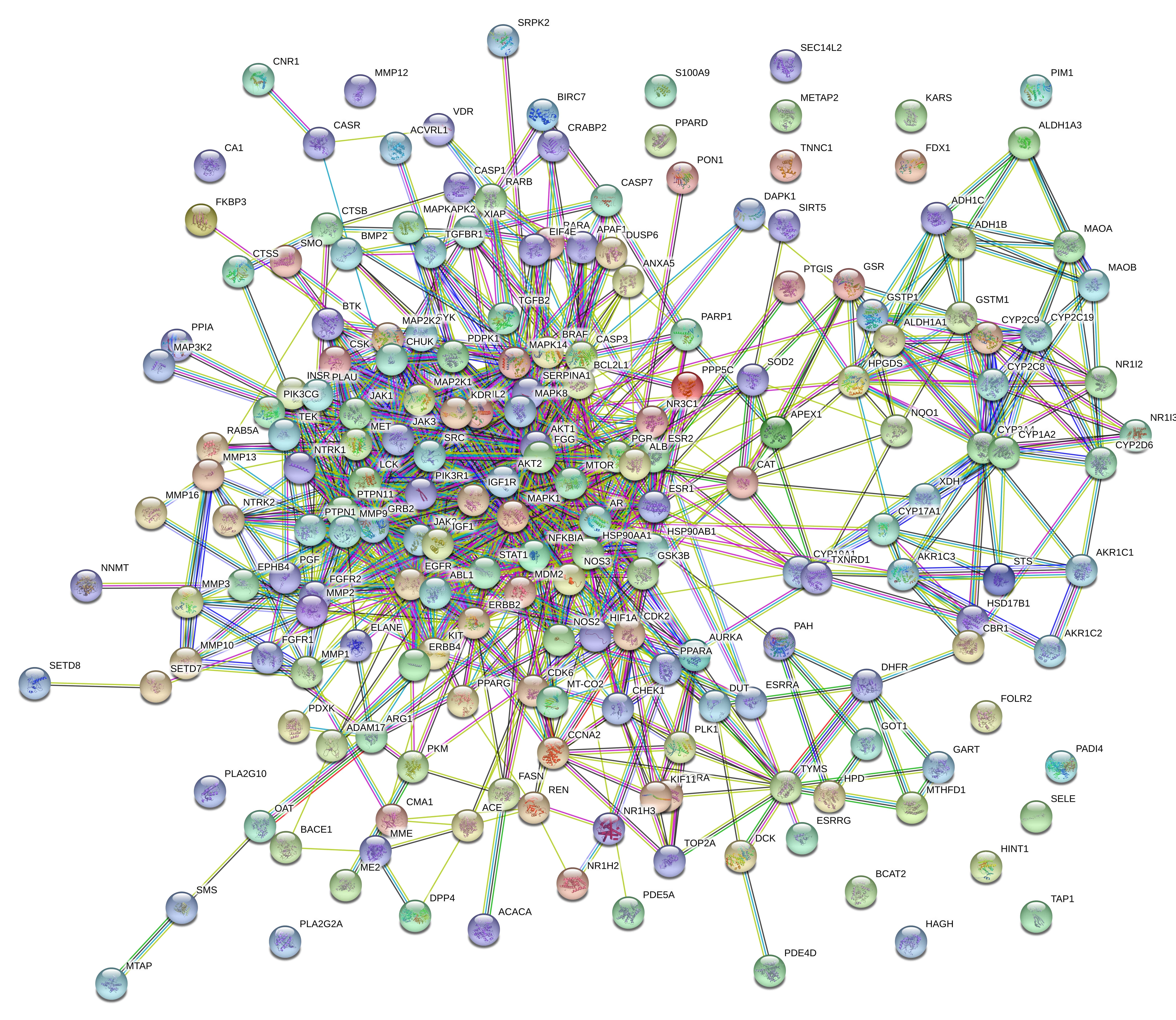

Supplement: Supplementary file 2 [file DataSheet2.ZIP › Supplementary Fig.2 (18a-GA STRING PPI network hugh resolution image).jpg]

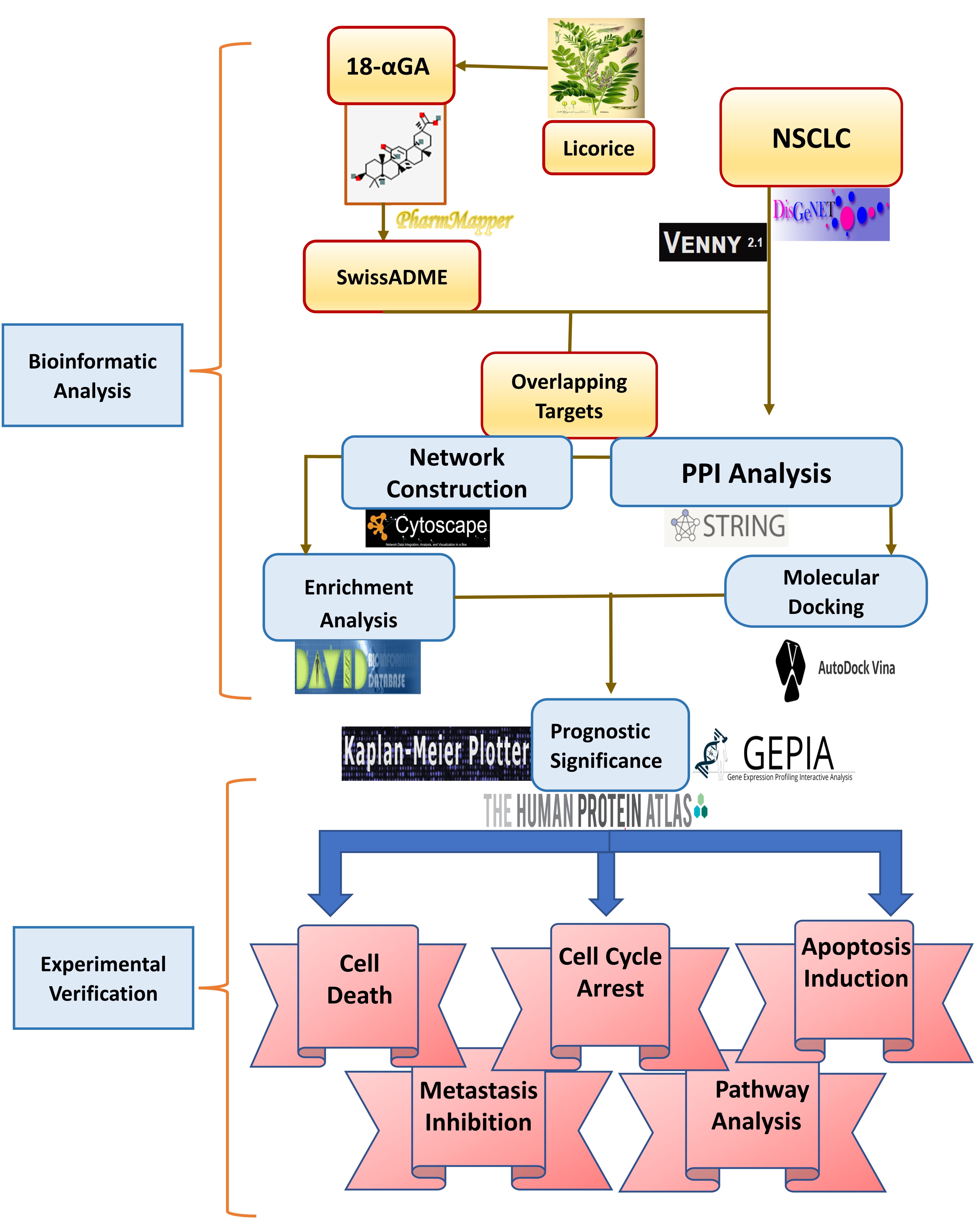

Supplement: Supplementary file 2 [file DataSheet2.ZIP › Supplementary Fig1 (overall framework of the study).jpg]

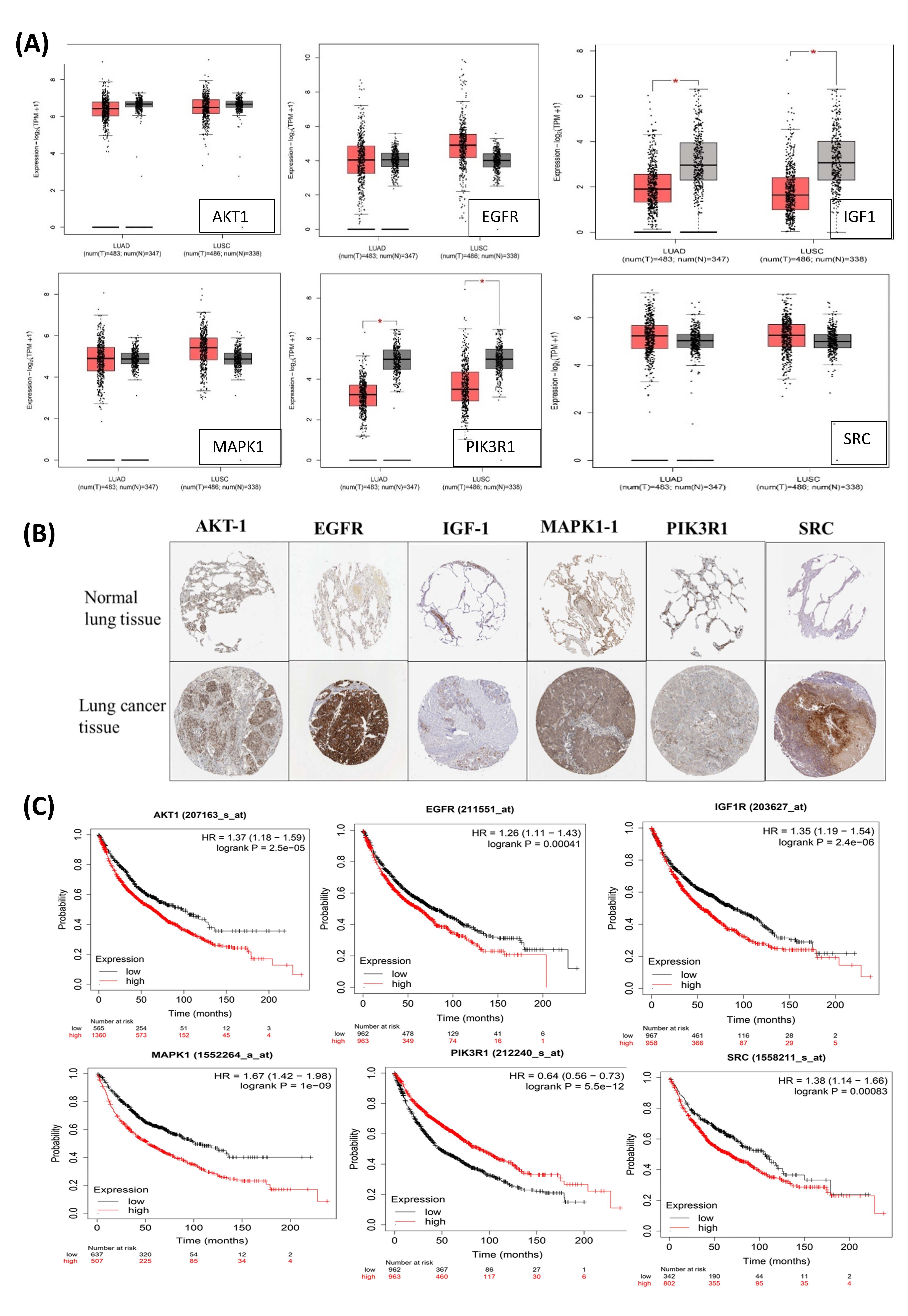

Supplement: Supplementary file 2 [file DataSheet2.ZIP › Supplementary Figure 3 (Survival analysis).jpg]
